# Choice of Illumination System & Fluorophore for Multiplex Immunofluorescence on FFPE Tissue Sections

**DOI:** 10.1371/journal.pone.0162419

**Published:** 2016-09-15

**Authors:** Sandrine Prost, Ria E. B. Kishen, David C. Kluth, Christopher O. C. Bellamy

**Affiliations:** 1 Department of Pathology, University of Edinburgh, Deanery of Molecular Genetics and Public Health Sciences, Queen's Medical Research Institute, Edinburgh, Scotland, United Kingdom; 2 Centre for Inflammation Research, Queen's Medical Research Institute, University of Edinburgh, Edinburgh Medical School, Edinburgh, Scotland, United Kingdom; 3 Department of Pathology, University of Edinburgh, Royal Infirmary of Edinburgh, Edinburgh, Scotland, United Kingdom; Consiglio Nazionale delle Ricerche, ITALY

## Abstract

The recent availability of novel dyes and alternative light sources to facilitate complex tissue immunofluorescence studies such as multiplex labelling has not been matched by reports critically evaluating the considerations and relative benefits of these new tools, particularly in combination. Product information is often limited to wavelengths used for older fluorophores (FITC, TRITC & corresponding Alexa dyes family). Consequently, novel agents such as Quantum dots are not widely appreciated or used, despite highly favourable properties including extremely bright emission, stability and potentially reduced tissue autofluorescence at the excitation wavelength. Using spectral analysis, we report here a detailed critical appraisal and comparative evaluation of different light sources and fluorophores in multiplex immunofluorescence of clinical biopsy sections. The comparison includes mercury light, metal halide and 3 different LED-based systems, using 7 Qdots (525, 565, 585, 605, 625, 705), Cy3 and Cy5. We discuss the considerations relevant to achieving the best combination of light source and fluorophore for accurate multiplex fluorescence quantitation. We highlight practical limitations and confounders to quantitation with filter-based approaches.

## Introduction

From beginnings in the early 20^th^ century, fluorescence microscopy has evolved to a widely used research and diagnostic tool capable of high quality image generation for the study of tissues. Fluorophores, illumination sources and optics continue to improve with the development of new dyes and hardware to meet demands from researchers. Investigators in cancer immunotherapy and tissue immunology now seek to discriminate and quantify multiple (5 or more) fluorophore labels applied to single samples, especially tissue sections, for *in situ* cell phenotyping. To achieve these goals with clinical formalin-fixed paraffin-embedded (FFPE) tissue samples, researchers must also address the challenge posed by auto-fluorescence. Many studies are based on fluorescence microscopy of tissue sections, but relatively little data is available critically evaluating the capacity and limitations of standard approaches compared with the range of newer methodological options, and in particular the potential benefits and limitations for multiplex tissue immunofluorescence.

Among newer fluorophores, the non-organic Quantum dots (Qdots) have highly favourable characteristics, outperforming traditional dyes such as FITC, TRITC or their more stable recent counterparts such as the Alexa dyes, in key metrics. These include brightness and photostability, a large Stokes shift and a broad choice of narrowband emission spectra across the visible spectrum into infrared [1–5] (S Prost Manuscript PONE-D-16-16178 under revision). The Qdots excitation spectrum also peaks around 400nm, which potentially reduces autofluorescence with FFPE tissue (see results). Nevertheless, while fluorophore performance is critical, the illumination system, labelling protocol and analysis technology also affect the quality of results. Traditionally, mercury light (HBO) with selectable excitation filters has been favoured for fluorescence microscopy as a powerful wideband source of excitation wavelengths across the visible spectrum to ultraviolet (UV). However, newer metal halide and LED systems lack the drawbacks of HBO that include explosive hazard, cumbersome alignment, non-uniformity, temporal instability and fading. Metal halide provides a similar excitation spectrum, but with stronger emission outwith the peaks of mercury light, is brighter overall, has controllable intensity, greater uniformity and stability [6]. New LED systems also offer uniform and dimmable illumination, typically of narrow band width, which is potentially advantageous for multi-colour fluorescence. LED emission is more intense than mercury for some, but not all wavelengths (for example green excitation spectra) [7].

In the present study, we consider the factors contributing to an optimal imaging system for multiplex immunostaining of human FFPE tissue, focusing on light sources, fluorophores and their interaction. We compare in detail the relative merits of 3 different light sources (HBO, metal halide and LED) and different fluorophores, including 7 Qdot labels, Cy3 & Cy5. We then further compare 3 different LED systems with a particular focus on stability of the fluorescence, autofluorescence and specificity of emission. We use spectral unmixing and image analysis to accurately determine the occurrence and magnitude of specific fluorescence, bleed-through and autofluorescence with different setups, which are otherwise not separately measurable in more conventional filter-based imaging systems. The findings are applicable to any system of image capture and analysis. We highlight key areas of consideration and potential weakness for investigators wishing to accurately measure multiplex-stained tissues, and recommend an approach to maximise the combined benefits of the different components.

## Materials and Methods

### Light Sources

Illumination sources tested were mercury light (HBO100 watts), metal halide (Olympus), Sola (Lumencor), CoolLED PE2 with 425 & 535/615 LAMs, Sola 2 (Lumencor, CoolLED PE4000) [8,9]. The Sola and Sola 2 produce LED-based white light continuous spectrum from 380 to 680 nm; by contrast the PE2 and the PE4000 produce selected wave lengths between 365nm and 770nm [8,9]. The PE2 unit can drive up to 4 LED wavelengths from 2 LAMs (LED Array modules) while the PE4000 has 16 selectable LED wavelengths which can be activated singly or in groups up to 4 [10]. The PE4000 can also be used as a source of white light but this was not tested here. Both companies provide alternative systems that were not tested. All excitation was done with the light source at full power unless specified.

### Microscope and Filters

The microscope used is an Olympus BX51, with a Chroma filter cube “Qdot set with long pass emission filter“(set 32013 Exciter E460SPUVv2; emitter E500lp; Dichroic 475dcxru). For the comparison of LED illumination sources in the 2^nd^ part of the study, the set was improved by changing the excitation filter with a Semrock FF01-417/60-25 (laser2000) (average transmission >90% over 60nm compared with 70% for E460SPUVv2) (S1 File). The data presented was captured using a x40 objective (Olympus UplanFl 40x/0.75 ∞/0.17); brighter objectives (40x Olympus UPlanSApo 40x/0.95 ∞/0.11–0.23/FN26.5 and 20x Olympus UPlanSApo 20x/0.75 ∞/0.17/FN26.5) reduced exposure times by 30–50% (S2 Table).

The Cy5 filter was Cy5-4040C (Semrock) and Cy3 filter Cy3-4040C (Semrock) adapted with a reduced band emission filter FF01- 543/22 (Semrock) to prevent bleed through).

### Multiplex Immunofluorescence and Fluorophores

We used the Labelled StrepAvidin Biotin (LSAB) method with streptavidin-conjugated Qdots for single colour staining or a combination of LSAB technique and indirect immunofluorescence with secondary Ab conjugated with Qdots (all Life technology). Briefly, 4μm FFPE sections of human liver or kidney were treated with *histosolve* (Fisher ThermoScientific) and rehydrated. Antigen retrieval was accomplished with 0.1mM EDTA pH8 in a microwave pressure cooker for 10 minutes at pressure before TBST washes. The sections were treated with the Vectorlabs avidin/biotin blocking kit (SP-2001) where appropriate and incubated with antiCD68 (macrophage marker, Dako, M0876) at 1/150 at room temperature for 45 minutes. After TBST washes, sections were incubated with biotinylated antimouse IgG3 (AbCam ab97258) 1/200 for 30 minutes, followed by incubation with streptavidin-conjugated Qdots (Life technology) for 30 minutes or with the appropriate secondary Ab conjugated with Qdot. Qdots were pre-diluted to give a comparable final intensity of positive fluorescence (preferable for quantification) (Table 1 & S1 Fig). Slides were then dehydrated, cleared, and mounted in Qmount (Life technology). For 5 color immunostaining (Qdots 525, 565, 655, Cy3 & Cy5) the first two fluorophore steps (Cy3 & Cy5) were completed on a Bond immunostainer (Leica) using *TSA plus* fluorescence system (NEL744 & NEL745 Perkin Elmer), according to the manufacturer’s instructions. The antigen retrieval step was performed with the *epitope retrieval 2* (Leica, EDTA pH8) and repeated after the Cy3 (Ki67) and the Cy5 (CD31) staining, followed with manual staining using an indirect method for Qdot 585 (CD163) and 655 (CD86) and a final streptavidin-Qdot 525 (CD68) step as previously described (S Prost Manuscript PONE-D-16-16178 under revision). TSA Alexa 488 & 655 (Thermofisher) were also tested, but the fluorescence intensity was too weak compared with Qdots and those fluorophores were not investigated further.

**Table 1:** Qdots molar extinction coefficient ε (cm^-1^M^-1^ determined at 405nm) and dilutions used to achieve a similar level of fluorescence (adapted from [11]).

Product & catalogue name	ε(cm^-1^M^-1^)	dilution
Qdot 525 Q10141MP	360,000	6/100
Qdot 565 Q10131MP	1,100,000	4/100
Qdot 585 Q10111MP	2,200,000	2/100
Qdot 605 Q10101MP	2,800,000	1/100
Qdot 625 A10196	9,900,000	1/750
Qdot 655 Q10121MP	5,700,000	1/200
Qdot 705 Q10161MP	8,300,000	2/100

Note that the there is a logarithmic correlation between **ε** and the chosen dilutions apart for Qdot 705 which requires a higher titter to achieved a similar level of fluorescence. This is likely due to the lower performance of the camera to detect emission in the infrared spectrum. (dilution = a-bxlog(**ε**) r = 097149 & r = 0.90376 without and with Qdot 705 respectively; see S1 Fig).

### Imaging and Analysis

**Fig 1:**
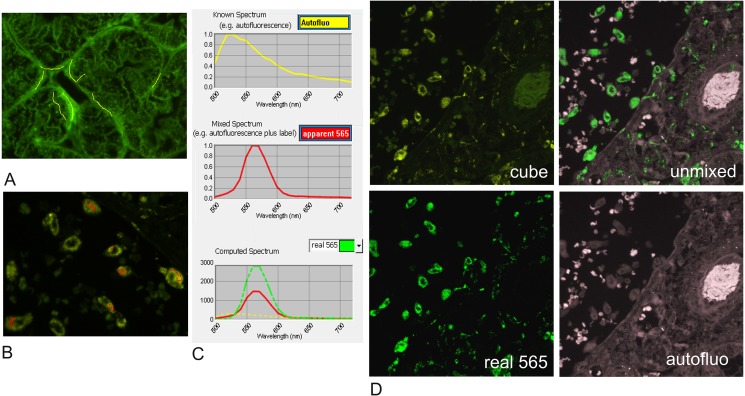
Example of spectral unmixing using Nuance [12,13]. FFPE tissue sections are imaged at single wavelengths every 10nm from 500nm to 720nm (an image “cube”). Unstained and single color stained control sections establish the specific emission spectrum of each color of interest and the auto-fluorescence spectrum (Fig 1A–1C). Those spectra are used to “unmix” grey-scale images from the cube dataset corresponding to the true intensity of fluorescence of each color of interest and separately the background autofluorescence in the test section. A) shows tissue autofluorescence whose spectrum in the regions under the yellow drawn lines on the image is depicted in the adjacent yellow spectrum plot. B) Similarly shows single colour staining for Qdot565 and the associated spectrum (red drawn lines and plot), which is a compound of fluorophore with autofluorescence. In C), the autofluorescence contribution to the staining spectrum for Qdot565 shown in B has been subtracted in Nuance, giving the actual specific staining intensity for the fluorophore. D) shows true intensity images computed in this way from the parent cube data, discriminating autofluorescence and colour marker components for each pixel, which can be viewed and quantified individually. Each fluorescence images are scaled using the Clip/Stretch option within Nuance mapping the lowest 0.01% of the pixels to 0, the highest 0.01% to 255, with linearly interpolation in between those values for visualisation.

Investigators often rely on filters to mitigate relative spectral contamination from auto-fluorescence by truncating the spectrum captured, but this approach has no effect on auto-fluorescence within the captured bandwidth. Using spectral unmixing [12] with a tuneable camera (Nuance FX camera, CRI; now PerkinElmer [13]) we separated the complete auto-fluorescence spectrum for individual wavelength analysis (Fig 1), allowing us to accurately compare the effect of different illumination sources on autofluorescence across the fluorophore emission spectrum, 500-720nm. Measurement data are Scaled Counts/Second which represents the counts after scaling for exposure time, binning, camera gain, and bit depth (Scaled counts/s = (counts/full scale) x (1/exp2) x (1/bin2)x (1/gain); with the full scale being 4096 for 12 bits acquisition. The value is therefore independent of those settings.

Various Worldwide Web based modelling tools are available to help match fluorophores, filters and illumination systems. We used Semrocksearchlight [14] to model the best fluorescence set-up for a 7 Qdots multiplex and obtain a theoretical comparison of illumination with mercury light, metal halide light, Sola LED white light and a series of single wave lengths LED illumination from COOL-LED. An example of theoretical data obtained for Qdot 605 is shown in Table 2. This was repeated for the 7 Qdots (S1 Table). From the theoretical analysis, PE2 LED system with a 405nm LAM was predicted to give the best fluorescence signal with a good signal to noise ratio for all the Qdots (Table 2 and S1 Table). Real comparisons were then performed using archival FFPE human liver and kidney tissues, known for their strong autofluorescence.

**Table 2:** Comparison of theoretical fluorescence parameters using semrocksearchlight.com; example of Qdot 605.

**Fluorophore**: Qdot 605					
**Filter Set:** Exciter FF01-405/150; Emitter FF01-500/LP; Dichroic FF510-Di02					
Light Source	405 LED Single Wavelength CoolLED	425 LED Single Wavelength CoolLED	Metal Halide 120mW	Lamp Mercury Arc Lamp	LED Broadband Lumencor SOLA
**Fluorescence Signal (mW)**	1	0.82	0.47	0.46	0.28
Excitation Light Noise (mW)	1	4.10	*	*	*
Autofluorescence Noise (mW)	1	1.01	0.49	0.51	0.37
Signal-to-Noise Ratio	1	0.81	0.96	0.90	0.77
A					
**Fluorophore**: Qdot 605					
**Filter Set:** Exciter FF01-405/150; Emitter FF01-500/LP; Dichroic FF510-Di02					
Light Source	405nm LED CoolLED pE-4000	435nm LED CoolLED pE-4000	Metal Halide X-Cite 120mW	HBO 100 Mercury Lamp	LED Broadband Lumencor SOLA light engine
**Fluorescence Signal (mW)**	1	0.79	0.493	0.555	0.414
Excitation Light Noise (mW)	1	1.16	22	171000	22.4
Autofluorescence Noise (mW)	1	1.01	0.501	0.527	0.518
Signal-to-Noise Ratio	1	0.785	0.984	0.217	0.8
B					

The test was performed for all Qdots, a selection of possible filters, and various available illumination sources. Example for Qdot 605 is shown compared with PE2 LED 405 & PE2 435nm (A) and compared with PE-4000 405 & 435nm (B) (data for the other Qdots in S1 Table)

LED 405nm is taken as reference. The Light Source Power (mW) was set at 100, Numerical Aperture 0.5, Index of Refraction 1, Reflected Excitation Light Factor 0.07, Transmitted Excitation Light Factor, 0.95, Fluorophore Optical Depth (M cm) 1.00E-12, Fluorophore DMAC (M^-1^cm^-1^) 80000, Fluorophore Quantum Yield 0.5, Autofluorescence Light Factor 0.1 and Transmitted Emission Factor 0.95

Test A was performed in 2014, test B in 2016.

*No data available

### Tissue Sourcing and Ethical Consent

The clinical tissue samples (inflamed human liver and kidney samples) used in this study were surplus archival paraffin block material originally procured for diagnosis. The archival tissue block samples were fully anonymised to the researchers and as such do not require patient consent. Their use for this study was fully approved by the local clinical governance and ethics committee (Lothian SAHSC and Tissue Governance Unit) with Approval Reference: 07/S1102/21.

## Results

### Initial Illumination Comparison for Qdot Fluorescence: Mercury versus Metal Halide versus LED

#### Metal Halide causes photobleaching of Qdots and strong autofluorescence of red blood cells

**Fig 2:**
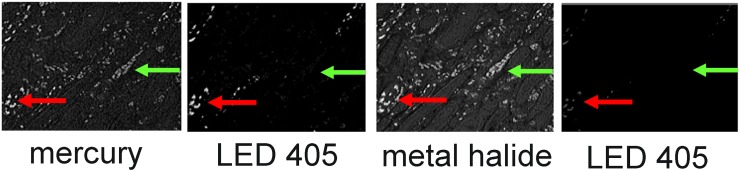
High levels of autofluorescence and fast photobleaching of specific fluorescence when illuminating Qdots with metal halide. FFPE section stained with Qdot 655 was imaged successively using mercury bulb, LED405 & Metal Halide source. The images are intensity images at 655nm. Red arrows: specific 655 fluorescence; green arrows: autofluorescence. The image taken with the first exposure to LED405nm illumination. (LED405 _(1)_) shows the specific staining with little autofluorescence when the section is illuminated with LED 405nm. Metal halide exposure led to high level autofluorescence and fast photobleaching of the specific fluorescence, confirmed with a second set of images with LED 405nm (LED405 _(2)_). Similar results were obtained with all other Qdots.

FFPE sections were labelled with Qdots and imaged successively using mercury light, LED 405 and Metal Halide source. Metal halide or mercury bulb illumination (HBO100W) of FFPE sections labelled with Qdots each produced more autofluorescence than did LED 405 (Fig 2). Furthermore, with metal halide illumination, photobleaching of Qdots was clearly appreciable with simple eye-balling. Photobleaching was still observed even with the illumination reduced to 10% intensity, so metal halide was discarded as inappropriate for quantification of Qdot intensity.

#### Mercury light provides good illumination of Qdots but higher autofluorescence

Mercury light illumination of Qdots also gave greater autofluorescence than LED 405, especially of red blood cells (Fig 2), but as no photobleaching was apparent by eye, further evaluations were made. Slides labelled with each Qdots were imaged using mercury bulb or CoolLED PE2 with either a 405 or a 425nm LAM (LED Array Module). Intensity images were generated by Nuance and the specific and background (autofluorescence) intensities were recorded.

**Fig 3:**
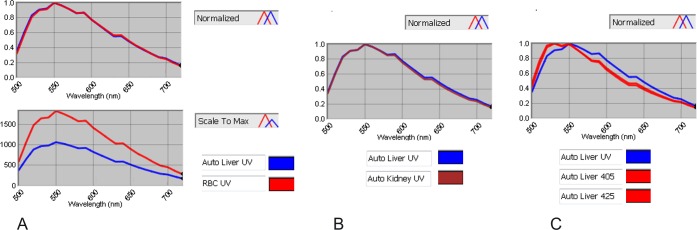
Comparison of the spectra of autofluorescence in FFPE liver, red blood cells (RBC) and FFPE kidney. A: spectra of red blood cells (RBC—red) and liver autofluorescence (AUTO Liver—blue) using a normalised scale (A top panel) and with unnormalised scale (A bottom panel). B: spectra for liver and kidney autofluorescence (AUTO Liver- blue; AUTO Kidney- red) using a normalised scale. C: spectra for liver autofluorescence excited by mercury light (blue) or LED 405 & 425 (both red). Autofluorescence of FFPE liver and RBC have a similar spectrum (A- top: overlapping curves) but different intensity (A bottom). FFPE liver and kidney have a similar spectrum of autofluorescence (B)–(A, B are shown for mercury light illumination; similar results were obtained with LED illumination). Autofluorescence spectrum of tissue illuminated with UV and LED are different (C). All were performed using the Qdot filter and autofluorescence spectra could be distinct with a different excitation range.

The autofluorescence of the tissue and associated red blood cells (RBC) had similar spectra for a given illumination source in both the liver (Fig 3A) and the kidney samples (Fig 3B). However, the autofluorescence spectrum under UV illumination differed from that with LED 405 & 425, having a peak of intensity around 525nm for UV compared with 550nm for LEDs (Fig 3C).

The overall intensity of autofluorescence between 500 and 720 nm was greatest in UV-illuminated samples (UV: 0.964 counts/s versus 0.135 and 0.146 counts/s in the same region of interest illuminated with LED 405 & 425nm respectively). However, this observation is not necessarily pertinent to researchers using filter blocks to evaluate only fluorescence around the emission peak of their specific fluorophore. We therefore measured the levels of autofluorescence and of Qdot fluorescence on images taken at specific wavelengths: serial sections were either unlabelled (autofluorescence test) or labelled with the same antibody and one of the 7 Qdots tested. The same areas from stained and unstained serial sections were illuminated using the different light sources and intensity images were capture for emission wave length corresponding to the peak of emission of each specific Qdot. The average specific fluorescence intensities (CD68 in macrophages) and autofluorescence intensities (in proximal tubules) were quantified using the Nuance analysis software. Table 3 gives the average intensities of autofluorescence and corresponding specific fluorescence recorded at the peak of emission of the selected Qdot (i.e. autofluorescence intensity at 525nm when stained with Qdot 525; autofluorescence intensity at 565nm with Qdot 565 etc.).

**Table 3:** Comparison of fluorescence intensities when illuminating Qdots with mercury light, LED 405nm or LED 425nm.

Mercury bulb				LED 405 nm			LED 425 nm		
Qdot	positive cells	autofluo	Ratio	positive cells	autofluo	Ratio	positive cells	autofluo	Ratio
**525**	1.557	1.183	1.317	0.566	0.201	2.819	1.289	0.158	8.158
**565**	1.483	1.177	1.259	0.329	0.170	1.940	0.445	0.177	2.517
**585**	1.342	1.107	1.213	0.497	0.152	3.270	0.419	0.154	2.715
**605**	3.466	0.888	3.904	0.545	0.118	4.624	0.615	0.117	5.273
**625**	4.252	0.697	6.097	0.436	0.091	4.805	0.764	0.094	8.139
**655**	2.032	0.537	3.786	0.898	0.069	13.032	0.875	0.067	13.094
**705**	0.852	0.266	3.205	0.305	0.011	27.869	0.250	0.010	23.396

The table gives the levels of fluorescence (average counts/s) in positive cells stained with the indicated Qdot (**positive cells**) and in the unstained control on a serial section (**autofluo**), under illumination by 3 systems (Mercury light, LEDs PE2-405nm & PE2-425nm). The levels of positive fluorescence and autofluorescence are recorded using the spectral camera with tunable filter at one unique wave length corresponding to the peak of fluorescence emission of each Qdot (ie 525nm for Qdot 525; 565nm for Qdot 565 etc).

The **Ratio** of specific fluorescence to autofluorescence (pink column) is an indication of the discrimination between autofluorescence and specific fluorescence: the higher the ratio specific:autofluorescence, the better. Note that LED 405 is generally a poorer exciter than LED 425.

Please note that the conditions used for the present test lead to a weaker specific signal than normally obtained with Qdots and were chosen to test the limits of the system.

Unlike suggested by the theoretical data (Table 2 & S1 Table), mercury light was a much stronger exciter of Qdots when compared with either LED source (compare the intensity of fluorescence levels in positive cells excited by mercury bulb with LED Table 3). However, the intensity of autofluorescence in surrounding tissue was also more intense under mercury bulb illumination (Table 3), leading to a much poorer overall signal-to-noise ratio compared with LED. The ratio for LED 425nm being over 2–7 fold greater than mercury bulb with different Qdots (Table 3 Ratio).

#### LED-425 gives better Qdot illumination than LED-405

**Fig 4:**
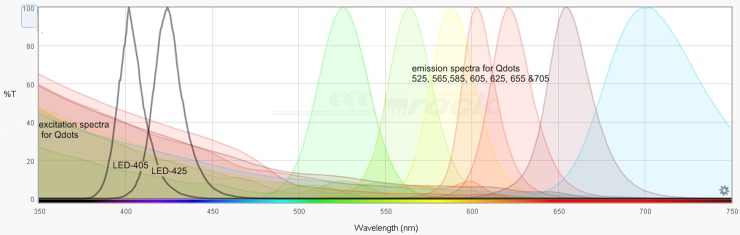
Excitation & emission spectra for Qdot fluorescence. The graphs show the theoretical excitation spectra and emission spectra for the Qdot fluorophores used in the study—adapted from SemrockSearchlight.com. Note that at shorter wave lengths the excitation should be better. The excitation filter used is allowing wave lengths from 350 to 460 with a slightly better transmission rate around 405 than 425 (ASCII data in [15]); the dichroic mirror is 475nm and the emission filter is a 500nm long pass (Chroma filter set 32013). The illumination spectra for LED-405 & LED-425nm are shown in black.

Interestingly, the excitation profile for Qdots (Fig 4) suggests that the lower wavelengths give greater excitation, as did the theoretical assessment using Semrock searchlight (S1 Table). However, we found that LED-425nm was a similar or better exciter than LED-405nm for 5 out of 7 Qdots (Table 3) and moreover that the specific:autofluorescence ratios were consequently better when using 425nm, especially for the green and yellow wave lengths (525nm & 565nm), where autofluorescence from FFPE is the strongest (Fig 3). Simultaneous illumination with 405 and 425nm LAMs was not better than LED-425 alone (data not shown). LED illumination therefore had clear advantages compared with mercury bulb and metal halide for fluorescence studies using Qdots in FFPE samples. In addition to excellent temporal and special stability, there was no significant photobleaching of the Qdots unlike with Metal Halide, and tissue autofluorescence was reduced compared with mercury bulb illumination.

#### Comparison of white light LED and single wavelength LED for multicolour fluorescence using Qdots with other fluorophores

Having initially settled on a single excitation system with 425nm (PE2-CoolLED) excitation which was found to be optimum for Qdots work, we investigated the possibility of performing multicolour fluorescence combining Qdots with other fluorophores. Commercial LED illumination systems are either white light equivalent or with selectable single colour emission diodes. Qdots share a common excitation spectrum (350-480nm, with emission between 525 and 800nm depending on the Qdot), but other fluorophores impose additional excitation requirements on selectable illumination systems, requiring additional LED Array Modules (LAM). For testing, we evaluated a modern white light LED source (Sola2), having improved power especially in green wavelengths, together with two selectable LED sources: firstly, the PE4000 from CoolLED, with capacity for 16 wavelengths that can be turned on individually or in groups of 4; secondly, the PE2 with 2 or 4 LAM. The Sola2 and PE4000 were tested with illumination of Qdots, Cy3, Cy5, Alexa 488 and 555, and further compared with the PE2 for Qdots, Cy3 & Cy5. Using spectral unmixing we analysed the overall fluorescence intensity between 500 and 720nm, the peak emission intensity and the relative level of specific and autofluorescence at the emission peak.

#### Intensity of autofluorescence and specific fluorescence with Qdots

**Fig 5:**
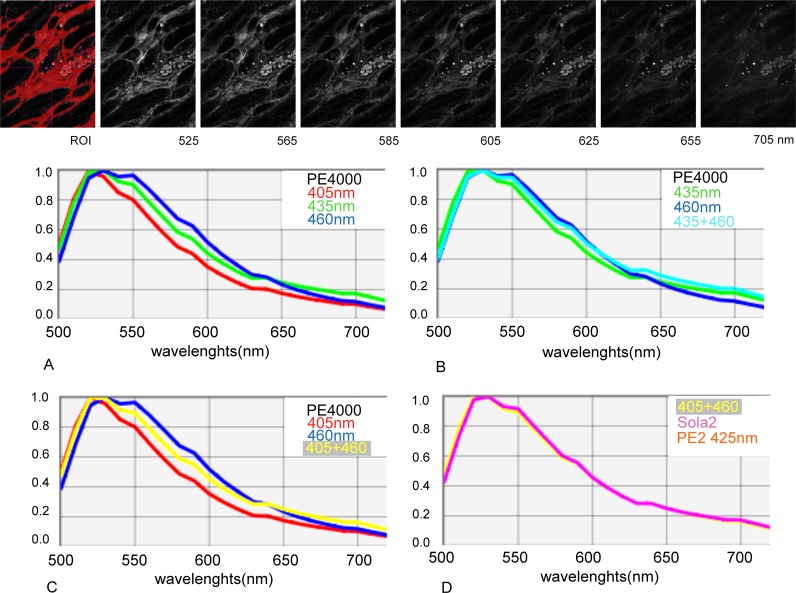
Comparison of the spectra of autofluorescence of FFPE kidney tissue under illumination with PE4000 at 405nm; 435nm 460nm or combinations; PE2 425nm or Sola 2. **Top panel:** An example of the autofluorescence intensity images under illumination with 405+460nm is shown for the indicated emission wave lengths which correspond to the peak of emission of the studied Qdots. ROI shows a typical thresholded region of interest selected for quantification of the intensity of autofluorescence. **Graphs**: The spectra of autofluorescence were found to be fairly similar between 500 and 720nm. However the level of autofluorescence between 530 to 650nm increases with the excitation wave length with PE4000 (A&C). SOLA2, PE2 425 & PE4000 405+460nm lead to similar autofluorescence spectra (D).

Illumination with each LED source, PE4000 (405+460nm), Sola2 and PE2 (425nm), gave similar autofluorescence spectra (Fig 5D). However, autofluorescence increased when illuminated at increasing wavelengths between 540 and 650nm with PE4000 (405<435<460 Fig 5A, 5B and 5C).

We quantified autofluorescence and specific fluorescence from Qdots 525, 565, 585, 605, 625, 655 and 705 on serial FFPE kidney sections labelled with the same specific primary antibody (Ab) against CD68 and a secondary Ab complexed with the indicated Qdot. Matching fields of the stained and unstained serial sections were illuminated with the indicated light source and fluorescence images were taken every 10 nm from 500 to 720nm using the Nuance tuneable camera. The exposure time was set to achieve 70% of the dynamic range (autoexposure function) and the exposure times were recorded (Table 4). The autoexposure time reflects the strength of excitation provided by each illumination system to each fluorophore as well as the level of autofluorescence where this is not negligible. It is dependent on the strength of the immunofluorescence (function of the extinction coefficient and quantum yield of each fluorophore as well as the level of the target labelled in the tissue), the autofluorescence of the tissue and the microscope configuration, including the optical characteristics of the objective.

**Table 4:** Autoexposure times to achieve 70% of the dynamic range.

Autoexposure time (ms)	PE2	Sola 2	PE 4000				
	425 nm	White LED light	405nm	435nm	460nm	405+460 nm	435+460 nm
Qdot 525	149.26	141.97	404.11	236.61	501.15	250.31	176.70
Qdot 565	71.20	69.22	213.18	122.14	311.52	137.70	86.78
Qdot 585	76.12	79.61	257.76	145.01	356.85	164.00	99.17
Qdot 605	273.6	265.3	541.392	365.376	994.624	1018.86	409.425
Qdot 625	55.14	55.95	178.83	88.66	224.56	100.12	68.08
Qdot 655	193.47	151.28	311.52	251.26	546.54	306.51	220.99
Qdot 705	252.08	335.94	1035.70	553.14	1609.26	865.58	508.94
autofluo	514.66	383.09	1609.73	737.09	2000.00	1029.58	467.90

A field of view of sections stained with the indicated Qdot was successively illuminated by all indicated light sources in the order indicated in the table.

The exposure time is automatically calculated by the Nuance system to prevent saturation of pixels by restricting the exposure time to 70% percentage of the dynamic range of pixel values. A second autoexposure value was recorded for PE2 before moving to another section stained with the next color to confirm the absence of photobleaching between the first and the last exposure (data not shown).

**Fig 6:**
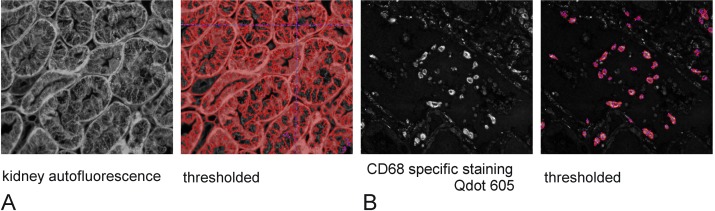
Autofluorescence and specific fluorescence threshold for quantification. Intensity images of FFPE kidney sections, unstained (autofluorescence) or stained with for CD68 using specific Ab and secondary conjugated with Qdot were quantified at the peak of fluorescence using Nuance software. This was performed for each Qdot, illuminated by each light source. The figure shows the example of the emission at 605nm for a slide labelled with Qdot 605 and an unstained section from the same tissue (autofluorescence) illuminated by LED4000 435nm. A: Intensity image of kidney tubules autofluorescence at 605nm, and corresponding thresholded areas for quantification of the autofluorescence intensity. B: Intensity image of a serial section labelled with Qdot 605 at 605nm and thresholded image for quantification (minimum 100 pixels connected).

**Fig 7:**
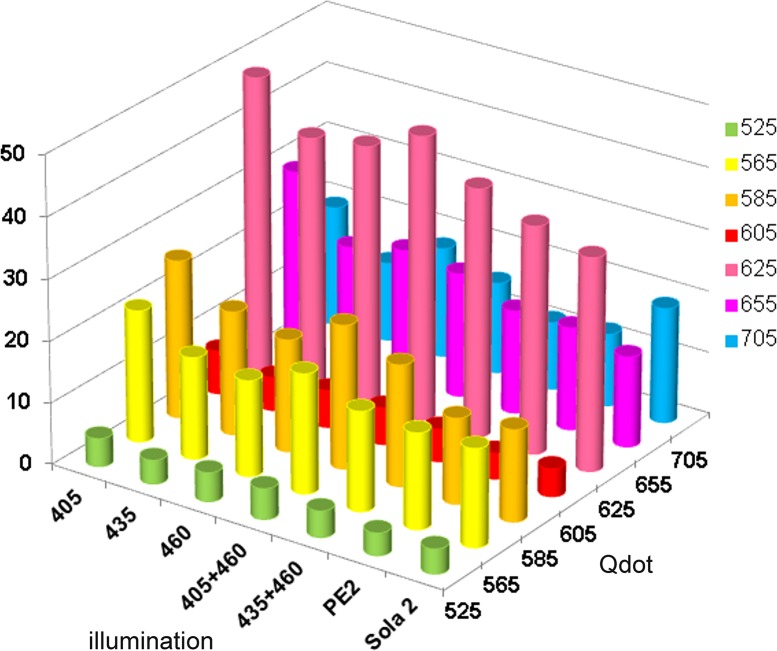
Specific fluorescence ratio when excitation Qdots various LED illumination system. The intensity of fluorescence at the peak of emission for each Qdot was recorded in macrophages labelled with antiCD68 and a secondary antibody conjugated with the indicated Qdot. The intensity of autofluorescence at the same wavelength was obtained from an unstained slide. The figure shows the specificity of fluorescence for each Qdot, illuminated with PE4000 at 405nm (405); 435nm (435); 460nm (460), simultaneous illumination with 405 and 460nm (405+460), 435+460 (435+460), PE2 and Sola2. The crude data are given in Table 5C.

The intensities of autofluorescence and of specific fluorescence were measured at the peak fluorescence for each fluorophore (Fig 6 and Table 5). Excitation with Sola 2 & PE2 gave the strongest autofluorescence (Table 5A) and specific fluorescence (Table 5B) for each fluorophore. However, the ratio of specific to autofluorescence was best for most fluorophores with the PE4000 405 nm (Table 5C & Fig 7).

**Table 5:** Autofluorescence and specific fluorescence emission.

**A**							
**Autofluo at**	**525 nm**	**565 nm**	**585 nm**	**605 nm**	**625 nm**	**655 nm**	**705 nm**
**405**	0.061 +/- 0.027	0.043 +/- 0.016	0.035 +/- 0.012	0.026 +/- 0.008	0.02 +/- 0.005	0.019 +/- 0.003	0.012 +/- 0.002
**435**	0.137 +/- 0.059	0.101 +/- 0.038	0.083 +/- 0.029	0.061 +/- 0.019	0.047 +/- 0.012	0.041 +/- 0.008	0.027 +/- 0.004
**460**	0.043 +/- 0.019	0.037 +/- 0.015	0.032 +/- 0.012	0.024 +/- 0.008	0.018 +/- 0.006	0.014 +/- 0.004	0.009 +/- 0.002
**405+460**	0.098 +/- 0.043	0.075 +/- 0.029	0.063 +/- 0.022	0.047 +/- 0.015	0.036 +/- 0.01	0.031 +/- 0.007	0.02 +/- 0.003
**435+460**	0.175 +/- 0.076	0.135 +/- 0.051	0.112 +/- 0.039	0.083 +/- 0.026	0.064 +/- 0.017	0.054 +/- 0.011	0.035 +/- 0.006
**PE2**	0.28 +/- 0.112	0.201 +/- 0.07	0.164 +/- 0.052	0.12 +/- 0.034	0.092 +/- 0.022	0.079 +/- 0.014	0.05 +/- 0.005
**Sola 2**	0.207 +/- 0.037	0.167 +/- 0.045	0.146 +/- 0.054	0.114 +/- 0.055	0.091 +/- 0.052	0.079 +/- 0.051	0.047 +/- 0.03
**B**							
**Specific fluo at**	525 nm	565 nm	585 nm	605 nm	625 nm	655 nm	705 nm
**405**	0.282 +/- 0.093	0.917 +/- 0.269	0.898 +/- 0.191	0.183 +/- 0.034	0.988 +/- 0.362	0.542 +/- 0.125	0.238 +/- 0.044
**435**	0.536 +/- 0.171	1.686 +/- 0.492	1.68 +/- 0.354	0.335 +/- 0.068	1.926 +/- 0.715	0.789 +/- 0.195	0.351 +/- 0.067
**460**	0.205 +/- 0.069	0.584 +/- 0.172	0.589 +/- 0.123	0.147 +/- 0.033	0.757 +/- 0.284	0.302 +/- 0.075	0.154 +/- 0.03
**405+460**	0.49 +/- 0.162	1.459 +/- 0.432	1.467 +/- 0.312	0.282 +/- 0.055	1.676 +/- 0.625	0.63 +/- 0.167	0.298 +/- 0.061
**435+460**	0.753 +/- 0.241	2.2 +/- 0.65	2.221 +/- 0.474	0.447 +/- 0.09	2.575 +/- 0.96	0.905 +/- 0.249	0.392 +/- 0.082
**PE2**	1.033 +/- 0.299	3.137 +/- 0.711	2.287 +/- 0.603	0.51 +/- 0.096	3.402 +/- 1.054	1.319 +/- 0.335	0.594 +/- 0.094
**Sola 2**	0.839 +/- 0.312	2.659 +/- 0.846	2.184 +/- 0.69	0.521 +/- 0.126	3.153 +/- 1.282	1.168 +/- 0.302	0.888 +/- 0.146
**C**							
**ratio**	**525**	**565**	**585**	**605**	**625**	**655**	**705**
**405**	4.60	21.51	25.79	7.10	48.24	29.22	19.62
**435**	3.91	16.63	20.18	5.48	40.92	19.36	12.92
**460**	4.74	15.64	18.32	6.12	42.12	21.46	18.02
**405+460**	5.02	19.52	23.43	6.05	46.31	20.25	14.93
**435+460**	4.31	16.28	19.77	5.38	40.45	16.83	11.09
**PE2**	3.69	15.63	13.95	4.27	37.07	16.78	11.89
**Sola 2**	4.06	15.97	14.93	4.57	34.67	14.78	18.80

A: intensity of autofluorescence at indicated wave lengths (average counts/s +/- sdv) as quantified in unstained kidney tubules (Fig 6A). red: worse levels of autofluorescence.

B: specific fluorescence emission at indicated wave lengths (average counts/s +/- sdv) as quantified in CD68 stained macrophages (Fig 6B). green: best levels of specific fluorescence.

C: ratio of specific emission over autofluorescence at indicated wave lengths.

The ratio gives the level of specific fluorescence at the peak of fluorescence of each Qdot.

Highlighted are the best ratios for each specific Qdot at peak of fluorescence. Note that the autoexposure time (with a long pass emission filter Chroma emitter E500lp) for Qdot605 is often high and close to that of the autofluorescence (Table 4) showing that the intensity of Q605 fluorescence in the stain is low, however, as the autofluorescence intensity around this wavelength is lower than in the green emission area (Fig 5) the specific fluorescence ratio is good (D) at this specific wave length.

**Fig 8:**
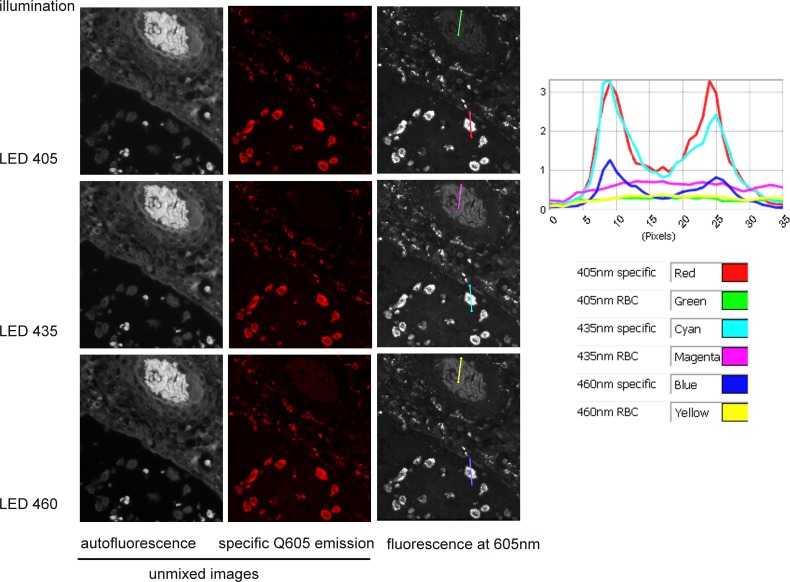
Comparison of specific and autofluorescence levels with LED illumination at 405, 435 or 460nm. Left & middle panels: intensity images unmixed from a cube of images taken between 500 and 720nm using a tunable filter camera (Nuance FX), showing the autofluorescence and Qdot 605. Right panel & curves: Images were taken at 605 nm (peak of specific fluorescence) using the Nuance tunable camera. The same profile lines drawn on red blood cells (green, magenta & yellow lines) and on a macrophage positively labelled (red, cyan & blue) give the fluorescence intensity profile shown on the graph. Note that specific staining is of similar level in pictures taken under 405 and 435 nm (red & cyan curves) but autofluorescence is stronger in 435nm (compare magenta & green curves).

When using spectral unmixing, increased autofluorescence is not an issue as it can be separated from the specific fluorescence (Fig 1) and a fast capture is beneficial. However, without access to spectral unmixing, the best specific:autofluorescence ratio should be sought–especially when dealing with low level target signal. The example given for Qdot 605 (Fig 8) shows that LED 405nm would be the best illumination option in that circumstance: the unmixed images (left and middle columns) and their intensity plot show the underlying composition of data that would be generated with simple epifluorescence image acquisition (right column): both autofluorescence (left column) and specific fluorescence (middle column) are visible contributors at the emission peak of 605nm, and this autofluorescence is particularly strong with 435nm illumination (magenta line). By contrast, the plot shows that illumination at 405nm gives a high specific fluorescence (red line profile) and the lowest autofluorescence (green line), while 460nm illumination has similarly low autofluorescence (yellow line) but lower specific fluorescence (dark blue line) (Fig 8 & Table 5C). Given that the target used for the study is a high density label (CD68 in macrophages) he data show that illumination quality is a sensitive determinant of signal dynamic range: suboptimal illumination wavelengths can render low density targets unreliable for quantification as signal levels approach closer to autofluorescence, generating a low dynamic range of quantifiable intensity. LED 405 here represents the best combination of bright specific fluorescence with low autofluorescence.

#### Illumination of Qdots in combination with other fluorophores

**Fig 9:**
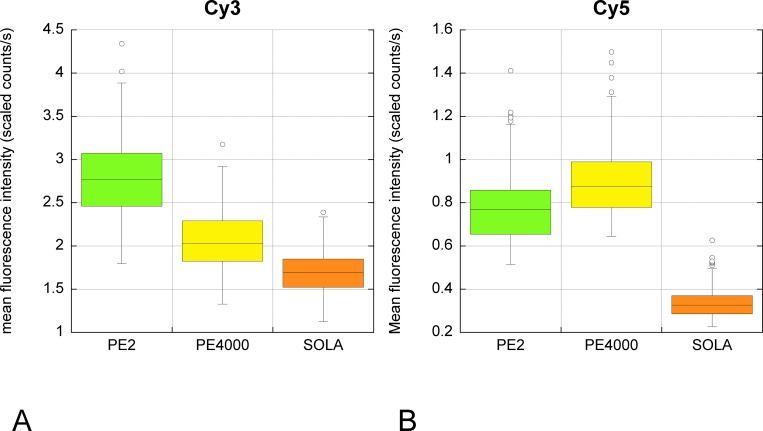
**Comparison of unmixed specific fluorescence Cy3 (A) & Cy5 (B) when excited by white light LED (SOLA), PE2 (535nm & 615nm) or PE4000 (555 & 635nm).** The same field of view illuminated with the indicated LED system was captured through the appropriate filter (for Cy3 & for Cy5) between 500 and 720nm. The fluorescence intensity (scaled counts/s) at emission peak wavelengths was obtained after spectral unmixing. The excitation peaks of Cy3 and Cy5 are 554 & 649 nm respectively. Despite the LAMs used with PE2 not being the optimum available, the results were better than with the white light system.

The possibility of using other fluorophores together with Qdots was tested with Cy3, Cy5, Alexa 488 and Alexa 555. We found that the intensities of Alexa 488 and Alexa 555 were too weak (even with tyramide amplification) compared with Qdots and the bleed through too contaminating—including bleed through of Alexa 488 into the Alexa 555 detection filter—to use for reliable quantification even with spectral unmixing (data not shown). In contrast, Cy3 and Cy5 gave good signal intensity. We compared the excitation using PE2 (535 & 615nm respectively), Sola white light, or PE4000 (555 and 635nm). Although the LAM 615nm is not optimum for excitation of Cy3, PE2 can host LAMs for only 4 different wavelengths, which restricted the choice. LAM 615nm was already used for imaging the Qnuclear Deep Red (LifeTechnology) counterstain in our Qdot multiplex staining, so this was tested for excitation of Cy5. In these conditions and despite the suboptimal excitation wavelength, PE2 and PE4000 were comparable and gave better excitation of Cy3 and Cy5 than Sola white light, which was less effective than the single intensity LAMS (Table 6, Fig 9).

**Table 6:** Autofluorescence and specific fluorescence at peak of emission for Cy3 & Cy5 excited by white light LED (SOLA), PE2 (535nm & 615nm) or PE4000 (555 & 635nm).

**Cy3 at 570nm**	**PE2 535nm**		**PE4000 555nm**		**Sola**	
	**BK**	**specific**	**BK**	**specific**	**BK**	**specific**
**average**	**1.989757**	**9.160652**	**1.588668**	**6.795557**	**1.327651**	**5.540776**
**sem**	**0.253373**	**0.182925**	**0.014931**	**0.123194**	**0.224278**	**0.105282**
**ratio specific/bk**	** **	**4.603906**	** **	**4.277519**	** **	**4.173367**
**Cy5 at 670nm**	**PE2 615nm**		**PE4000 635nm**		**Sola**	
	**BK**	**specific**	**BK**	**specific**	**BK**	**specific**
**average**	**0.272805**	**0.919322**	**0.26374**	**1.122252**	**0.128934**	**0.52169**
**sem**	**0.01203**	**0.022824**	**0.025742**	**0.02747**	**0.010783**	**0.014581**
**ratio specific/BK**		**3.369884**		**4.255147**		**4.046169**

The specific staining was quantified in thresholded positive cells and 3 regions of interest were drawn manually in unstained tissue for quantification of the background fluorescence (BK).

The average fluorescence intensity (counts/s) was quantified at the peak of fluorescence on images taken at the appropriate wave length by the Nuance camera with Tunable filter (ie 570nm for Cy3 & 670nm for Cy5).

Peaks of excitation for Cy3 & Cy5 are 554nm & 649nm respectively.

**Fig 10:**
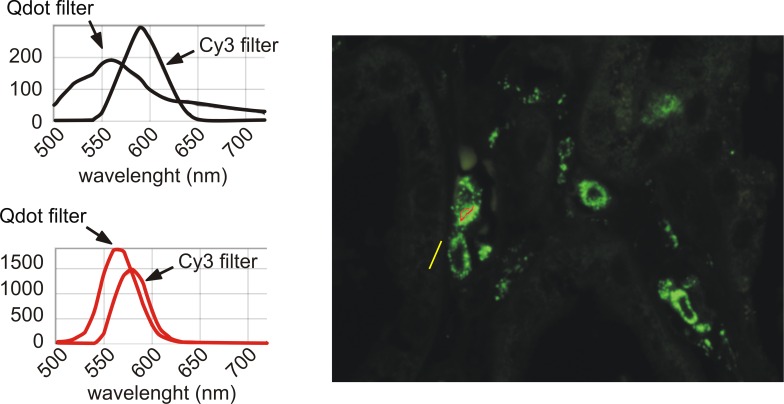
Significant bleed through of Qdot 565 in Cy3 filter when illuminated with white light LED. A slide stained with Qdot 565 was imaged with Nuance through Qdot filter and Cy3 filter using SOLA white light illumination. The specific and autofluorescence spectra (left panels) are obtained by sampling the appropriate image areas (ie positive cell for specific fluorescence: red line & curves; background for autofluorescence: yellow line & black curves) on the cube of images containing images at 10 nm intervals between 500 & 720nm though both filter cubes (right panel). As expected the spectra through the 2 emission filters are different (Cy3 emission filter 593/40; Qdot emission filter 500LP) as the Cy3 filter should pass wavelengths from 567 nm semrock [16], while the Qdot filter is a long pass filter from 494nm Chroma (see ASCII data in [15])

We next tested for confounding excitation/emission interactions among the fluorophores under illumination relevant to a multiplex imaging setting. Sections stained with one per section of the 7 study Qdots or Cy3 or Cy5 were each illuminated with all the wave lengths together that would be potentially used in a multiplex staining involving Qdots, Cy3 and Cy5. The resulting emitted fluorescence (specific, autofluorescence, or bleed through of specific fluorescence) was visualised directly through the microscope eyepieces and scored semiquantitatively (Table 7). Qdots under single wavelength excitation (PE4000 & PE2) gave a clear signal in the Qdot filter (in Table 7; green cells = good level of specific fluorescence) without any apparent bleed through across the Cy3 or Cy5 filters (in Table 7; black cells represent no fluorescence going through a given filter; all Qdots excited by all single wave length LEDs are “black cells” for Cy3 and Cy5 filters). Illumination of Qdots with the white light LED also gave no apparent bleed through across the Cy5 filter, apart from Qdot 655 (Table 7, row “Qdot655/Sola”; column “Cy5 filter” is colored pink for non expected fluorescence). By contrast, all Qdots under white light LED illumination showed specific fluorescence bleed-through across the Cy3 filter (pink cells for all “Qdots/Sola” in “Cy 3 filter” column), sometimes accompanied by autofluorescence (Table 7, S2 Fig). This bleed-through was particularly strong from Qdot 565, having a similar order of magnitude to fluorescence seen with the specific Qdot filter (Table 7, Fig 10). This effect may be due to the small absorbance peak for Qdot565 at 545nm (S3 Fig). Illumination with the single LEDs 405, 435 or 460nm does not activate Cy3, but the Sola white light LED can still illuminate and excite Qdots due to the passband within the Cy3 excitation filter between 500 & 550nm (S4 Fig). The emission peaks of Cy3 and Qdot 565 are close together, while Qdot 585 emission is fully passed by the emission filter for Cy3 (S3 Fig & S4 Fig). This then causes the observed bleed through of both Qdot 565 and 585nm emissions across the Cy3 filter under white light (Table 7). Conversely, Cy3 illuminated with the appropriate LED single wave lengths (PE2 & PE400 535nm) was excited with no bleed-through across the other filters (green for expected fluorescence in Cy3 filter, and black in other filters). However, illumination of Cy3 by white light LED or any single LED wave length suitable for Qdot excitation led to visible fluorescence bleed-through the Qdot filter (Table 7, pink rows in Qdot filter column). In these conditions, quantification of a multiplex using Qdots and Cy3 would not be possible without spectral unmixing (see further). These findings differ from the software modeller’s prediction that more bleed-through would occur with PE2 or PE4000 illumination than with Sola white light illumination (Table 8). Cy5 excitation by any of the light sources did not bleed through Qdot or Cy3 filters, as predicted (Table 7).

**Table 7:** Bleed through test.

Dye	Illumination	Cy5 filter	Qdot filter	Cy3 filter
**Qdot 525**	**PE4000 405nm**			
	**PE4000 435nm**			
	**PE4000 460nm**		+	
	**PE2 425nm**			
	**Sola**			AUTO
**Qdot 565**	**PE4000 405nm**			
	**PE4000 435nm**			
	**PE4000 460nm**			
	**PE2 425nm**			
	**Sola**			+++ (**)
**Qdot 585**	**PE4000 405nm**			
	**PE4000 435nm**			
	**PE4000 460nm**			
	**PE2 425nm**			
	**Sola**			++
**Qdot 605**	**PE4000 405nm**			
	**PE4000 435nm**			
	**PE4000 460nm**			
	**PE2 425nm**			
	**Sola**			++
**Qdot 625**	**PE4000 405nm**			
	**PE4000 435nm**			
	**PE4000 460nm**			
	**PE2 425nm**			
	**Sola**			+ & AUTO (***)
**Qdot 655**	**PE4000 405nm**			
	**PE4000 435nm**			
	**PE4000 460nm**			
	**PE2 425nm**			
	**Sola**	+		AUTO
**Cy5**	**PE4000 635nm**			
	**Sola**			
	**PE2 615nm**	(*)		
	PE2 425nm			
**Cy3**	**PE 4000 535nm**			
	PE4000 405nm		++	
	PE4000 435nm		++	
	PE4000 460nm		++	
	**Sola**		++	
	**PE2 535nm**			
	PE2 425nm		++	

Serial sections stained with the indicated dye (column 1) were illuminated with the indicated light (column 2) and of the observation of specific fluorescence (+/++/+++) and autofluorescence (AUTO) going through each filter block (Cy5; Qdot & Cy3 filter blocks) was recorded.

+/++/+++ reflect the intensity of the specific fluorescence; AUTO reflects the presence of noticeable autofluorescence.

**grey cells:** no visible fluorescence; **green cells:** expected specific fluorescence, strong unless indicated; **pink cells**: bleed through (i.e. observation of fluorescence in a set up where fluorescence is not desirable).

Excitation wave lengths that would not normally be used for a dye are indicated by **a red cell**.

(*) more autofluorescence was observed with PE2 615nm than with Sola & PE4000 635nm

(**) see Fig 10;

(***) see image in S2 Fig

**Table 8:** Theoretical bleed through for Qdot 565 in Cy3 filter, using semrocksearchlight.com.

**Theoretical data**	**single wave length LED**			**LED white light**		
Name	Qdot565 in Qdot filter	Cy3 in Cy3 filter	Qdot in Cy3 filter	Qdot565 in Qdot filter	Cy3 in Cy3 filter	Qdot in Cy3 filter
Filter Set	specific	specific	bleed through	specific	specific	bleed through
Exciter	FF02-435/40	FF01-543/22	FF01-543/22	FF02-435/40	FF01-543/22	FF01-543/22
Emitter	FF01-496/LP	FF01-593/40	FF01-593/40	FF01-496/LP	FF01-593/40	FF01-593/40
Dichroic	FF482-Di01	FF562-Di03	FF562-Di03	FF482-Di01	FF562-Di03	FF562-Di03
Fluorophore Emission	Qdot 565	Cy3	Qdot 565	Qdot 565	Cy3	Qdot 565
Light Source	CoolLED pE4000			Lumencor SOLA light engine		
	435 nm	525 nm	525 nm			
Fluorescence Signal (mW)	2.71E-06	3.54E-06	1.86E-07	7.60E-07	8.39E-07	4.22E-08
Excitation Light Noise (mW)	1.29E-08	2.72E-09	2.72E-09	2.15E-08	1.49E-09	1.49E-09
Autofluorescence Noise (mW)	0.0000325	0.0000034	0.0000034	0.00000937	0.000000747	0.000000747
Signal-to-Noise Ratio	0.083442	1.039	0.054611	0.080983	1.1212	0.056396
**Ratio single wave length /LED white light**		Qdot565 in Qdot filter	Cy3 in Cy3 filter	Qdot in Cy3 filter		
		specific	specific	bleed through		
Fluorescence Signal (mW)		3.565789474	4.219308701	4.407582938	**(1)**	
Excitation Light Noise (mW)		0.6	1.825503356	1.825503356	**(2)**	
Autofluorescence Noise (mW)		3.468516542	4.551539491	4.551539491	**(3)**	
Signal-to-Noise Ratio		1.030364397	0.926685694	0.968348819	**(4)**	
			**Single wave length**	**LED white light**	**Ratio single wave length/white light LED (7)**	
Bleed through signal Qdot/specific signal Qdot **(5)**			0.06863	0.05553	1.23607	
Bleed though into Cy3 / specific Cy3 **(6)**			0.05254	0.0503	1.04462	

From the theoretical data, ratio between the data given for single wave length and the white light illumination were calculated. They suggest that:

(1) The fluorescence signal would be stronger with single wave length LED but this would lead to more bleed through too

(2) The light noise for Qdot would be better with single wave length LED but worse than the white light illumination for Cy3

(3) The autofluorescence for Qdot would be worse with single wave length LED for Cy3 and Qdot

(4) The signal to noise ratio would slightly better for Qdot with single wave length LED but worse for Cy3

(5) The equivalent of 6.86% of Qdot specific fluorescence would be observed through Cy3 filter with single wave length LED & 5.55% with LED white light

(6) The equivalent of 5.25% of Qdot specific would go through Cy3 filter with LED white light & 5.03% with LED white light

(7) There would be more bleed through with single wave length LED, about 1.2 time more than with white light LED

**Fig 11:**
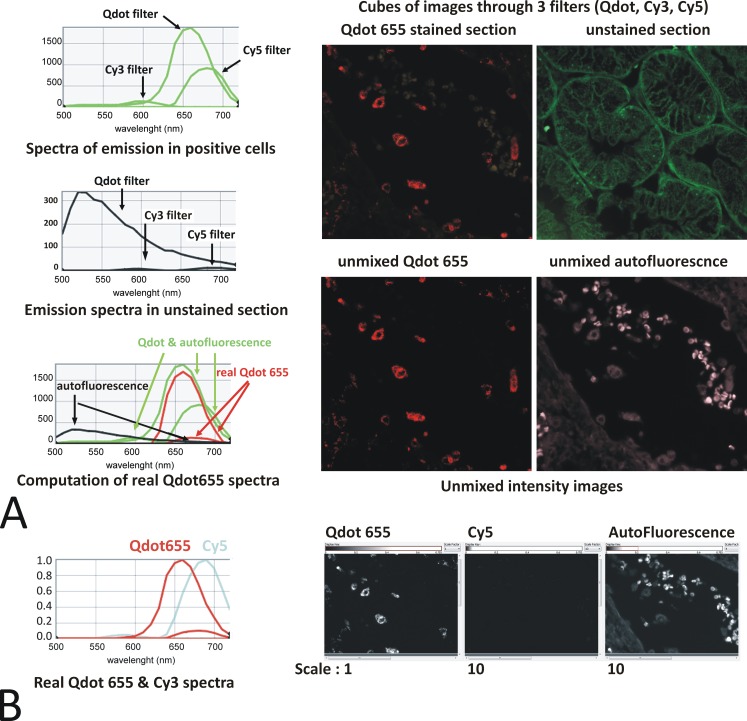
Qdot 655 imaged under Qdot, Cy3 & Cy5 conditions. **Evidence of bleed through in Cy3 filter and resolution with spectral unmixing.** A: A Qdot 655 single color immunofluorescence and an unstained kidney section were imaged with LED single wave lengths PE 4000 435, 550 & 635nm through the Qdot, Cy3 & Cy5 filter set respectively. The resulting cubes of images are shown in the top panel. The spectra of emission in the positive cells (Qdot 655 + autofluorescence–green curves) and the autofluorescence (black curves) were obtained, and the real Qdot655 was mathematically computed (red curves) using the Nuance software [13]: A peak of emission remains in the real Qdot spectra corresponding to genuine bleed through of Qdot 655 in the Cy5 filter. Note the presence of fluorescence in the Cy5 filter suggesting bleed through (the curve highs are dependent upon the exposure time of the image and thus do not reflect the intensity of the fluorescence). The Nuance spectral analysis software uses the spectral information from all 3 filters used to unmix a real Qdot 655 intensity image from the autofluorescence (bottom panel). B: A Cy5 stained section was used to similarly compute the real Cy5 spectra when imaged through the 3 filters (cyan curves). When this spectra, together with the real Qdot655 (red curve) & autofluorescence spectra are used to unmixed a Qdot655 single stained section, appropriately, no fluorescence is assigned to Cy5: Spectral unmixing can resolve the bleed through that a normal fluorescence system would not. Note the autofluorescence and Cy5 intensity images have been scaled up 10 fold to help visual assessment of the absence of fluorescence.

**Fig 12:**
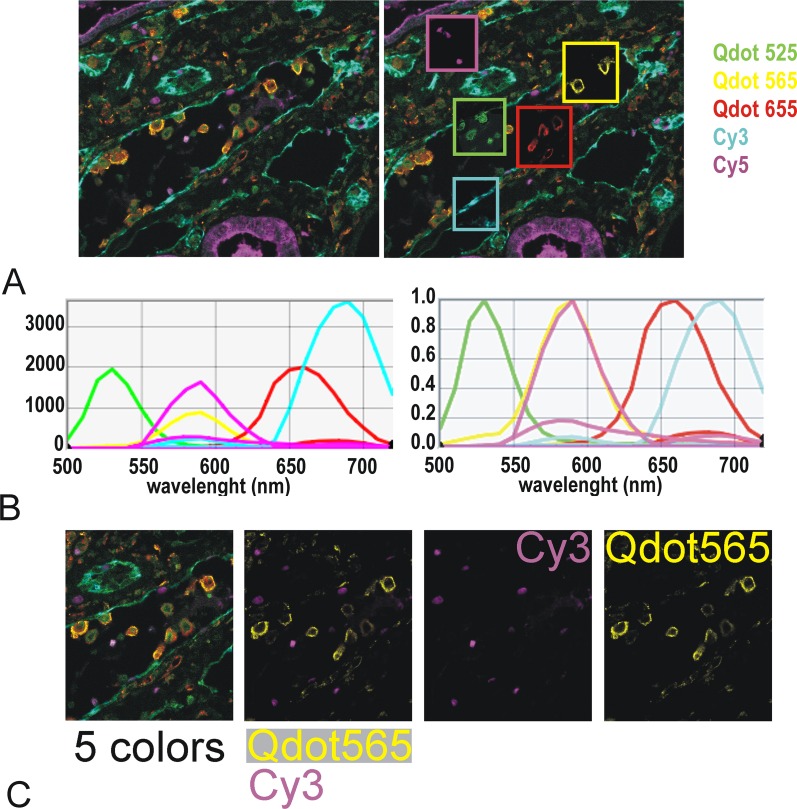
5-color multiplex using Qdot525, 565, 655 & Cy3 & Cy5. The stained FFPE section, illuminated with PE4000 single wave lengths (LAMs 435nm, 550 &635 nm) was imaged using Nuance Fx tunable camera between 500 & 720 nm through Cy3, Cy5 & Qdot filter cubes. The cube of images was unmixed according to single color spectra. A: left: unmixed image with all makers superimposed; false colors as indicated; right: same unmixed image showing in windows the individual unmixed markers. B: spectral library used for the unmixing. Left: scaled to max; right normalised. The resulting spectra take into account the emission of each fluorophore in each cube. The intensities of the dyes are of similar levels with Cy3 being about twice as bright as the other dyes (left). Note that although Cy5 & Qdot 585 have a similar peak of emission (yellow & magenta–right panel) Qdot 565 does not bleed through Cy3 filter (2 magenta curves for emission of Cy3 in both the Qdot & the Cy3 filter but only one yellow curve for emission of Qdot 565 in Qdot filter). C: cube (5 colors) and unmixed images showing an excellent unmixing of Cy3 (Ki 67 staining in few nuclear cells) and Qdot565 (CD68 staining in macrophages)

From direct visual inspection it was therefore clear that white light LED, but apparently not single wavelength LED, produced visible bleed through in many cases. However, measuring with the Nuance multispectral camera, we found that bleed through was also present with single LED illumination (Fig 11) albeit at a much lower level. Spectral unmixing systems like Nuance [13] can account for bleed-through and provide real intensity images (see example of a 5 color multiplex using 3 Qdots together with Cy3 & Cy5 in Fig 12), because the intensity is calculated according to the entire spectrum of each fluorophore. By contrast, the present data shows that quantification of this type of mixed fluorophore multiplex staining would be jeopardised with systems relying on filter blocks only.

## Discussion

We aimed here to identify the best system for accurate discrimination and quantification of fluorescence signals from immuno-detection of multiple antigens in FFPE tissue sections when using epifluorescence and a standard microscope. The present data explore in detail the interaction of light source, different fluorophores and tissue autofluorescence; they identify favourable combinations of different fluorophore types (Q dots and tyramide-amplified Cy3 & Cy5), while other combinations were found unsuitable (Qdots with Alexa fluorophores).

The ideal fluorophore would be bright, stable, without bleed through, with its excitation wavelength causing minimum autofluorescence at the emission wave length(s). Compared with conventional organic fluorophores, Qdots fluorophores have highly favourable physical properties for multiplexing studies, including high extinction coefficients and quantum yields, long fluorescence life time and stability [1] (Prost Manuscript PONE-D-16-16178 Under revision), as well as large Stokes shifts with narrow bright symmetrical emission spectra [2,11,17]. The present data validate the utility of those qualities in practice and confirm Qdots as well-suited to multiplex immunofluorescence study of tissue sections.

Bleed-through, for example of green into red [18], is an important confounder of epifluorescence evaluation of multiplex staining. In particular, even low percentage bleed-through of a bright fluorophore to a channel whose target is only weakly positive can obscure the latter with false signal. Mitigation strategies include attempting to equalise signal strengths in multiplex staining by careful combination of fluorophore, antibody concentrations and labelling technique. Varying illumination strength within an experiment is best avoided if possible but can ameliorate strong fluorophore bleed-through, as can reducing excitation filter bandwidth (e.g. from 531/40 for QnuclearDeepRed/Cy3 to 543/22-25, to reduce bleed through of Qdot 585 or 605). Nevertheless, filters including band-pass filters are not 100% efficient and may not be sufficient. The present data show that changing white light sources for monochromatic LED much reduces some instances of bleed through but that even so, measurable contamination can remain, detectable after spectral unmixing. Moreover, some fluorophore combinations such as Cy3 with Qdots were not remediable even with this strategy. The Qdots narrow and symmetrical emission spectra were an optimal solution here to prevent the problems of bleed-through, and although conventional fluorophores such as Alexa dyes were too weak in our setting to use alongside Qdots, tyramide amplification allowed effective combination with Cy5, if needed. Combination with Cy3 is also possible, when analysis is done with spectral unmixing.

Autofluorescence is another major confounder of accurate evaluation of fluorescence signals, especially with FFPE clinical samples [19]. Mammalian tissue autofluorescence derives from flavin coenzymes, reduced pyrimidine nucleotides, reticulin fibres, collagens and elastin as well as lipofuscin, to which is added fluorescence induced by formalin fixation, ubiquitous to standard clinical biopsy samples [20]. The level of endogenous fluorescence varies between tissues, being strong in liver and kidney. We found here that illumination source and wavelength has an impact on autofluorescence, with an increase between 560 and 680nm when using mercury bulb, LED 460 or Sola white light. This may in part reflect increased excitation of tissue flavins (excitation 360–520; emission 500–560) [21,22]. An understanding of the autofluorescence spectrum in the tissue of interest allows the investigator to select fluorophores with excitation and emission properties that minimise its impact, especially for detection of low density targets. Here, we found Qdots highly favourable compared with organic fluorophores such as Alexa 488, due to both reduced autofluorescence with the low wavelength excitation requirement of Qdots and because of the brightness of the Qdots.

We found that the choice of illumination source affected the performance of Qdots and other fluorophores: metal halide caused photobleaching of Qdots (and other fluorophores–data not shown) at such a rate that quantification of multiplex staining was not reliable in our configuration of manual capture of cubes of images for spectral unmixing. However, in a setting without spectral unmixing, others have successfully used rapid scanning detection of multiplexed Qdots under metal halide illumination [23]. When considering excitation, the light source would ideally match the excitation profile of the fluorophore without superfluous “contaminating”wavelengths. Broad spectrum light can be passed through carefully chosen filter sets, but the filter efficiency is never 100% and bleed-through of nonspecific wave lengths can significantly degrade the reliability of epifluorescence data, as observed here. “Monochromatic “LED reduce the likelihood of cross-illumination and autofluorescence compared with broad spectrum illumination (metal halide, mercury or LED white light), allowing a high contrast image with high signal-to-noise ratio. The outstanding temporal stability of the LED illumination compared with gas discharge lamps that need time to reach an equilibrium and decay throughout their life and which intensity fluctuates during use [24,25] is a further advantage for quantification over project lifetimes. However, the bandwidth of “monochromatic” LED varies from 10 to 85nm (example for PE -4000 [8]) so appropriate filters may still be required. Good Web-based tools are available to predict the performance of illumination, fluorophore and filter combinations and to estimate bleed-through and fluorescence intensity [14,26–29]. These provide a good starting point and acquaint the user with parameters that affect fluorescence quality. However, the estimates provided do not replace validation through practical testing: we observed significant discrepancies in fluorescence intensity and bleed-through with different light sources. Discussion with the technical representative for SemRock highlighted that Semrocksearchlight [14] was, at the time, assuming equal intensity for all CoolLED modules (personal communication). It is likely that the better excitation provided by PE2 LED 425 compared with PE2 LED 405 reflected different intensity of the 2 LAMs; such issues are to be expected with constantly evolving hardware iterations. Most vendors are willing to loan lights, cubes and other hardware for users to test before purchase (we had loans from Cool LED, Olympus, Semrock, Leica, Sola/ Lumencor for this study and from CRI and Zeiss previously) and we would recommend on-site testing after theoretical analysis.

Of course, other technical considerations not primarily the focus of this study also contribute to achieving a bright, specific and reliable signal [30,31], including configuration of the filter set (S1 File), microscope objectives (S2 Table), camera and the immunohistochemical detection method. There are several detection methods for successful multiple immunostaining, each with particular merits [18,32–35]. We have had success with different methods individually (indirect method, ABC, and tyramide amplification), and in combination (indirect with Qdots together with ABC with Qdots, and tyramide amplification with Cy3 & Cy5). Factors affecting choice of method include the nature of the target, including density and variability. The methodologic requirements to establish and maintain reproducible reliable staining are exacting [18,36]; they include standardised sample preparation (fixation, processing, section thickness, optimal antigen retrieval), [33] consistent positive and negative controls with each experiment, including for reproducibility, effect of the staining order, saturation and cross reactivity tests. Nevertheless, with a good labelling technique controlling for cross binding, the major limitations to quantification of multiplex fluorescence are the stability and uniformity of the fluorescence, confounding autofluorescence and bleed through, which are key outcome measures considered in this study. We have found Qdots illuminated with LED greatly superior in all these areas and would recommend their use with monochromatic LED. Broad spectrum LED can be more powerful and advantageous for qualitative fluorescence, or possibly where autofluorescence is not an issue (e.g. cultured cells) (high specific fluorescence emission of all Qdots illuminated by Sola in Table 5), but do not match the specificity and flexibility of monochromatic LED in this setting. We tested here lights from Lumencor (Sola broad light) and CoolLED (monochromatic), but both manufacturers make white light (broad spectra) and individually selectable LEDs. We found the material we tested from both companies to be excellent, with excellent technical and customer support. We therefore recommend a multi-monochromatic LED illumination system, but not a specific brand.

The present study shows that workable epifluorescence solutions to quantifying multiplex fluorescence can be achieved with careful planning, but the best solution to accurate quantification involves spectral unmixing, which accounts for bleed-through and autofluorescence. We use the Nuance FX system [12,13], but various techniques have been used both for fluorescence [23,37–39] and chromogenic stains [32,40,41]. Attempting quantification without spectral unmixing depends on a relatively crude “subtraction of background fluorescence”, which precludes reliable quantification of low level targets in particular. Our analyses of fluorescence when using the recommended filter cubes showed that autofluorescence or bleed-through were often so close to specific fluorescence (because of the typically restricted emission filter bandwidth) that they could only be discriminated using a camera with tunable filter and spectral unmixing with a long pass emission filter (tested for Cy 3, Cy5, Alexa 488, Alexa555 through specific single, double and triple band pass conventionally used in qualitative multicolour fluorescence). However, capture of cubes of images for multispectral analysis is time consuming: multiple pictures have to be taken at 10-20nm intervals across the emission bandwidth of each cube, which we like to be wide to obtain well defined spectra of emission. We have found this to benefit from the very bright staining that Qdots deliver, without needing over-amplification steps that easily saturate. It is possible to combine multiple single LAMS to increase the excitation intensity (435+460nm for excitation of Qdots for example); if this improves specific fluorescence without compromise from autofluorescence (Table 5) the reduced capture time may justify the compromise [31], especially if the fluorescence can be spectrally unmixed. Binning (S5 Fig) improves signal-to-noise ratio to the detriment of resolution, although we have not found it necessary with Qdots and LED.

In conclusion, we believe that with good wet lab protocols and controls, Qdots fluorophores (525 565 585 605 625 655 & 705), used alone or together with selected organic fluorophores, in a system using selectable monochromatic light LED and analysed with a spectral unmixing system is the system of choice for quantification of multiplex immunostaining on FFPE tissues. Such an approach is sensitive and specific, including for FFPE tissues considered particularly problematic such as liver and kidney.

## Supporting Information

S1 Fig Optimisation of streptavidin Qdot dilutions. (PDF)

S2 Fig Bleed through of Qdot 625 and autofluorescence with white light LED. (PDF)

S3 Fig Excitation of Qdots 565 & 585 through Cy3 filters. (PDF)

S4 Fig Qdot excitation leads to Cy3 bleed through in Qdot filter. (PDF)

S5 Fig Binning. (PDF)

S1 File Improvement of filter cube. (PDF)

S1 Table Comparison of theoretical fluorescence parameters using semrocksearchlight.com. (PDF)

S2 Table Improvement of objectives. (PDF)
