# Supplementary material for: Choice of Illumination System & Fluorophore for Multiplex Immunofluorescence on FFPE Tissue Sections
Source: PLoS One. 2016 Sep 15;11(9):e0162419. doi: 10.1371/journal.pone.0162419 (PMC5025086; doi:10.1371/journal.pone.0162419)
Supplement: S1 Fig — (PDF) [file pone.0162419.s001.pdf]

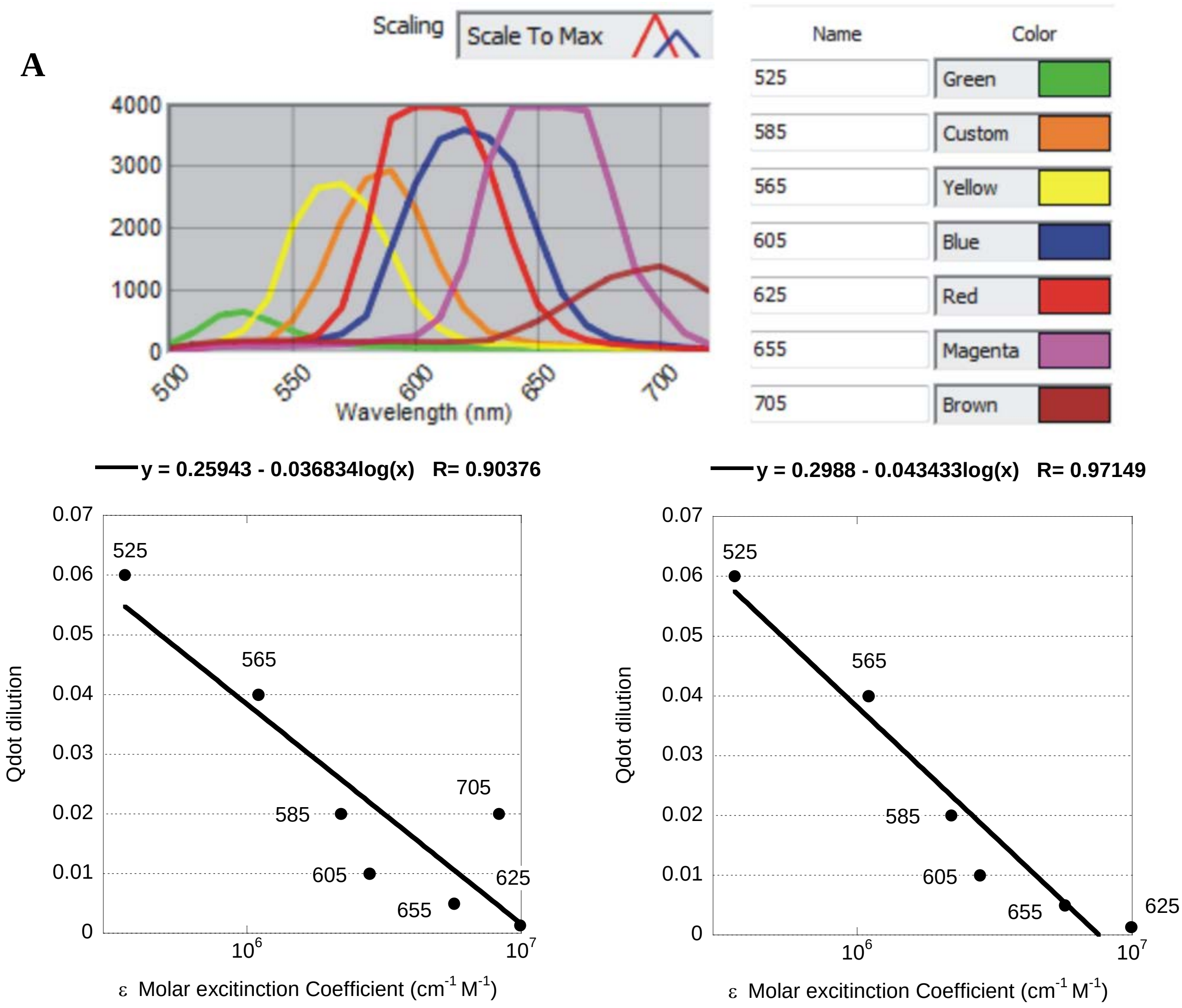

B **C**

S1 Fig: optimisation of dilution of streptavidin Qdot to obtain a fluorescence of similar intensity for all the Q dots

A : Immunofluorescence staining of CD68 was performed on the tissues of interest using the 6 different Qdots. Images were captured to evaluate the level of fluorescence of each fluorophore based on an autoexposure for Qdot 585 known to have an intermediary fluorescence level. This figure shows the relative level of intensity (scaled to the max.) for each Qdots in positive macrophages. Qdots 625 & 655 are shown here to be saturating (flat peak in red & purple) while the intensity for Qdot 525 was much lower than the other fluorophores. The dilutions were adjusted accordingly to get a more uniform intensity of staining and the final concentrations used are given in table 1

B & C : relationship between the chosen dilution and the molar extinction coefficient. There is a logarithmic correlation between ε and the selected dilutions, apart for Qdot 705 which required a higher titre to achieve a similar level of fluorescence. This is likely due to the lower performance of the camera to detect emission in the infrared spectrum. (dilution=a-bxlog(ε) r=097149 & r=0.90376 without and with Qdot 705 respectively).
