# Supplementary material for: Choice of Illumination System & Fluorophore for Multiplex Immunofluorescence on FFPE Tissue Sections
Source: PLoS One. 2016 Sep 15;11(9):e0162419. doi: 10.1371/journal.pone.0162419 (PMC5025086; doi:10.1371/journal.pone.0162419)
Supplement: S2 Fig — (PDF) [file pone.0162419.s002.pdf]

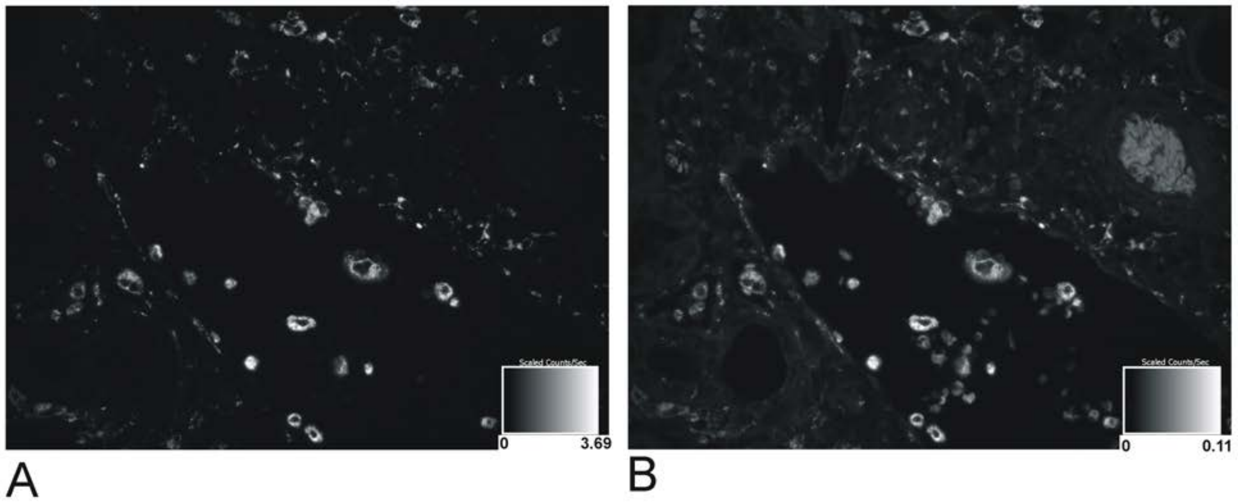

Fig : Bleed through of Qdot 625 & autofluorescence of Qdot625 stained slide illuminated with white light LED Sola

Sola illumination of a Qdot625 stained slide observed through (**A**) Qdot filter and (**B**) Cy3 filter

In the Cy3 filter, both autofluorescence & Qdot specific fluorescence is observed – this is only about 1/50th of the Qdot fluorescence level observed in the Qdot filter, but could be of similar intensity level to a specific Cy3 stain.

The scale shows the intensity of the images in scaled counts /s; clip/stretched similarly to allow for comparison

Note the autofluorescence of RBC and the specific Qdot staining in **B**
