# Supplementary material for: Choice of Illumination System & Fluorophore for Multiplex Immunofluorescence on FFPE Tissue Sections
Source: PLoS One. 2016 Sep 15;11(9):e0162419. doi: 10.1371/journal.pone.0162419 (PMC5025086; doi:10.1371/journal.pone.0162419)
Supplement: S3 Fig — (PDF) [file pone.0162419.s003.pdf]

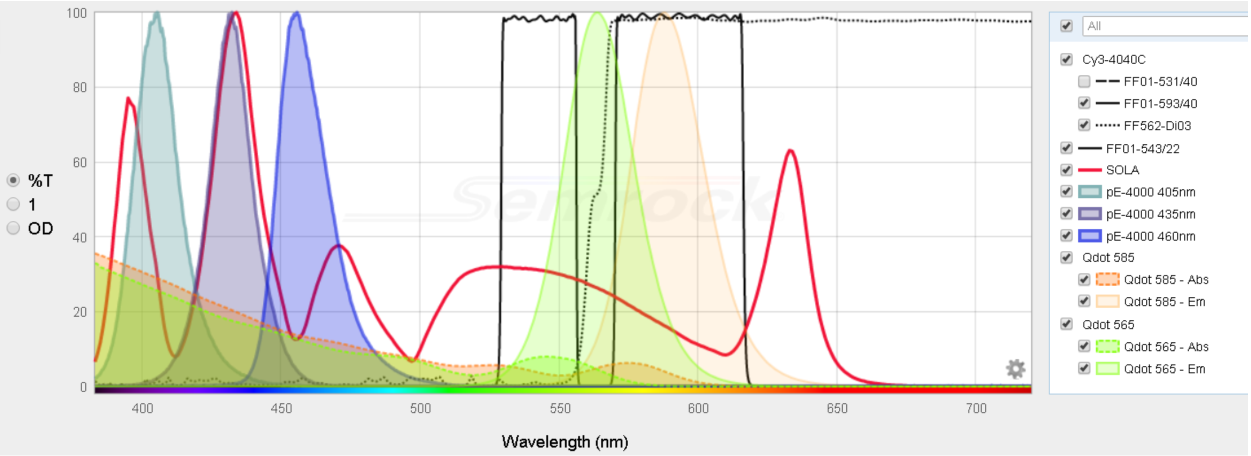

Fig : Excitation of Qdots 565 & 585 through Cy3 filters

The graph was created using SemRock searchlight.

Single wave length LEDs 405, 435, 460nm (various blues) do not go through the Cy3 emission filter (FF01-531/40 black line) while the white light Sola (red line) does, activating Qdots and leading to cross bleeding (emission shown here for Qdot 565 – green & Qdot 585 orange) of Qdots into Cy3 filter (Table 7).
