# Supplementary material for: Choice of Illumination System & Fluorophore for Multiplex Immunofluorescence on FFPE Tissue Sections
Source: PLoS One. 2016 Sep 15;11(9):e0162419. doi: 10.1371/journal.pone.0162419 (PMC5025086; doi:10.1371/journal.pone.0162419)
Supplement: S4 Fig — (PDF) [file pone.0162419.s004.pdf]

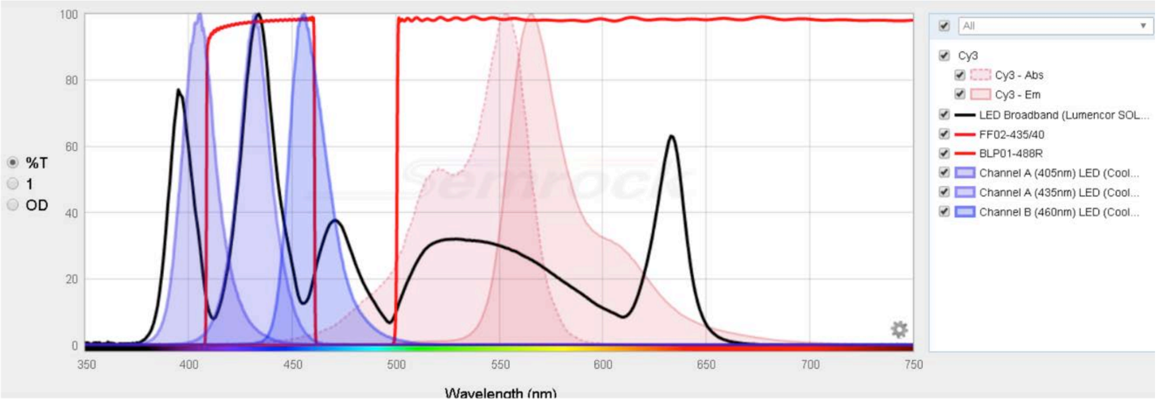

Fig : Qdot excitation leads to Cy3 bleed through in Qdot filter

The graph was created using SemRock searchlight.

Although the theoretical graph shows that Cy3 absorbance is limited (1 to 3 % between 440 & 460nm) within the Qdot excitation filter (FF02-435/40 red) all 3 single wave length LEDs 405, 435, 460nm (purple) as well as white light Sola (black line) were able to activate Cy3 to a non-negligible bleed through in Qdot emission filter level visible by eyes (Table 7).
