# Supplementary material for: Choice of Illumination System & Fluorophore for Multiplex Immunofluorescence on FFPE Tissue Sections
Source: PLoS One. 2016 Sep 15;11(9):e0162419. doi: 10.1371/journal.pone.0162419 (PMC5025086; doi:10.1371/journal.pone.0162419)
Supplement: S5 Fig — (PDF) [file pone.0162419.s005.pdf]

Pixel binning combines the information of adjacent pixels in a CCD camera sensor to create one single pixel in the captured image (1). This allows improvement of the signal to noise ratio, decreases the exposure time (about ¼ for a 2x2 binning) minimising the risk of photobleaching . However this is done to the detriment of resolution.
We tested the effect of binning on quantitative analysis of a 4 color immunostaining. As expected a reduced exposure time (about 1/4) was needed with 2x2 binning to achieve a similar autoexposure condition (not shown). After unmixing with the Nuance software the intensity images (scaled counts/s ie fluorescence count levels scaled for the effects of exposure time, binning, camera gain, and bit depth) were analysed using a custom AxioVision software.
The mean intensities of fluorescence of all 4 fluorophores was higher in the images taken with 2x2 binning, with a tighter spread of data (see box plot).

Brown et al (2) suggest that optimum pixel size for visible light with an objective with a numerical aperture greater than 0.8 is 0.1-0.2 µm. With the objective used (x40 NA0.95) the pixel size was 0.24 µm for 1x1 binning and an excellent resolution was achieved. In the present case, because of the brightness of the fluorophore and their stability in the appropriate conditions (S Prost Manuscript PONE-D-16-16178 under revision) either conditions can be used : 1x1 binning gave excellent resolution of the cells of interest, while 2x2 binning improved the of capture and the spread of data. Analysis of low expression marker would benefit 2x2 binning but in the present case we privileged the high resolution of the cell for more accurate selection of the cells of interest.

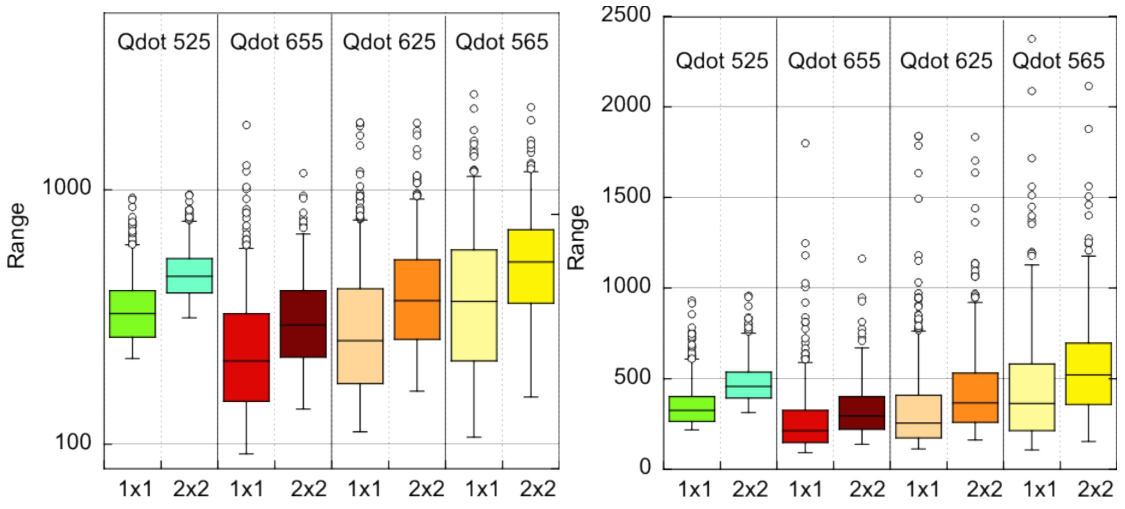

Fig : Binning

1. Abramowitz MD, M.W. Pixel binning. Olympusmicro.com2012 [cited 2016]; Available from: http://www.olympusmicro.com/primer/digitalimaging/concepts/binning.html.
2. Brown CM. Fluorescence microscopy–avoiding the pitfalls. Journal of Cell Science. 2007;120(19):3488-.
