# Supplementary material for: Choice of Illumination System & Fluorophore for Multiplex Immunofluorescence on FFPE Tissue Sections
Source: PLoS One. 2016 Sep 15;11(9):e0162419. doi: 10.1371/journal.pone.0162419 (PMC5025086; doi:10.1371/journal.pone.0162419)
Supplement: S1 File — (PDF) [file pone.0162419.s006.pdf]

Improvement of Filter cube

4 Filter combinations were compared : the Chroma filter cube “Qdot set with long pass emission filter” (set 32013 Exciter E460SPUVv2; emitter E500lp; Dichroic 475dcxru) (**Figure A - A**) and the Semrock filter (Bright line long-pass set exciter FF02-435/40-21.8-D FF01-500/LP-23.3-D FF510-Di02-22x29) (**Figure A -B**) set, both recommended by the respective companies as best for Qdots ; and the Chroma set where the emission filter was replaced either with the emission filter from the Semrock set (FF02-435/40-21.8-D), or another with a wider excitation range extending towards the wave lengths (FF01-417/60) (**Figure A - D**). Both recommended sets have their advantages : the excitation filter from Chroma look poorer in transmission but allowed illumination at lower wave lengths where Qdots can be efficiently excited. However, having observed that with that filter set the LED 425nm was a better exciter in our settings; the Semrock recommended excitation filter looked a better option, with a better percentage of transmission level. As for the emission, the filter in the semrock set appeared to cut out Qdot525nm (compare **A & B** in **Figure A**).

Both Semrock excitation filter (FF02-435/40-21.8 &FF01-417/60) have better transmission than the Chroma Filter (**S6 Fig1 C & D**).

Serial sections were stained with the same Ab and labelled with all 7 Qdots (525,565,585,605,625, 655,705). Pictures of the same field, illuminated with LED 425 were taken successively with the indicated filters. The intensity of fluorescence of the positive cells was analysed using Nuance software (**Figure B**).

All new filter combinations (**B, C, D**) improved the level of detected fluorescence for all Qdots compared with the Chroma filter already in use (**A**) (**Figure B & Table B**). Overall filter **B** was slightly better than the other 2, however the combination **C** gave the best results for Qdot 525, which is the Qdot with the lowest molecular extinction coefficient and brightness. We therefore choose to replace the excitation filter for FF02-435/40-21.8-D (Semrock) in order to improve detection of all Qdots, and more particularly of Qdot 525.

The graphs are adapted from the original Chroma filter graphs (https://www.chroma.com/products/sets/32013-qdot-set-with-longpass-emission-filter) and SemrockSearchLight graphs (https://searchlight.semrock.com/)

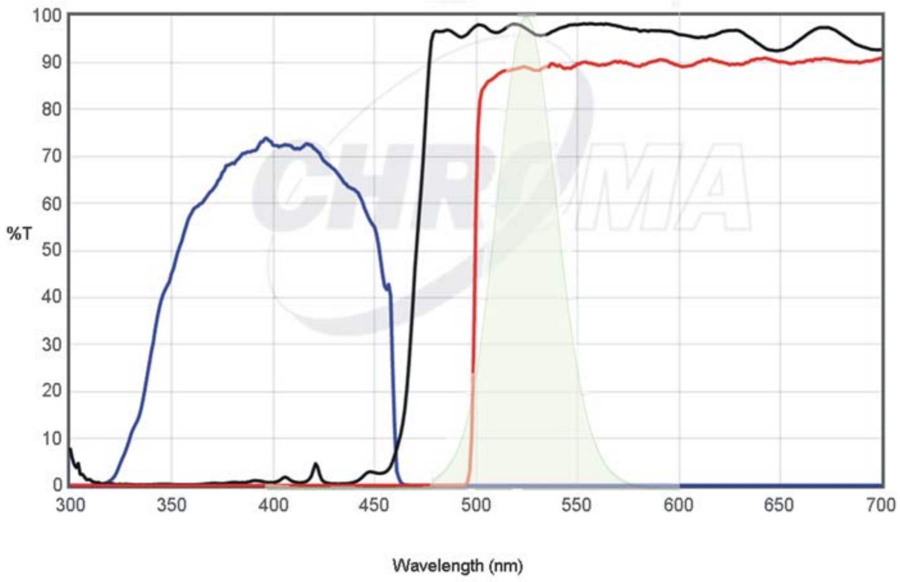

A

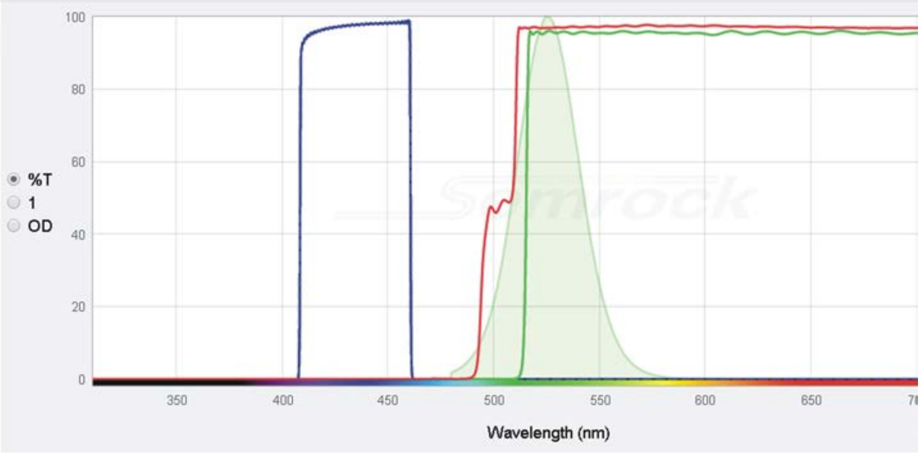

B

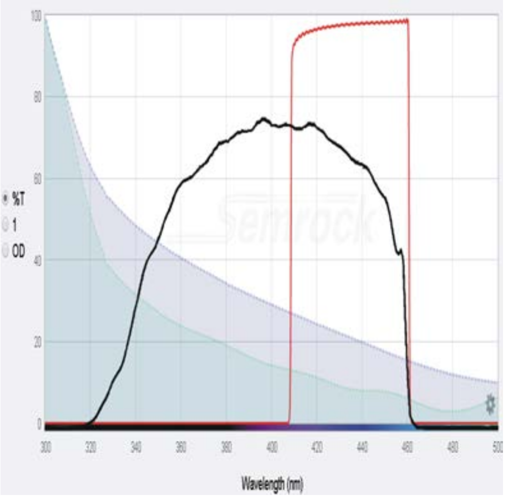

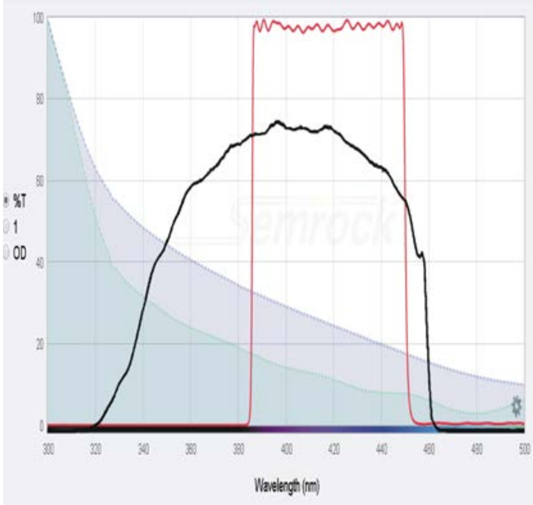

C **D**

Figure A : **Improvement of Filter cube - Filter Comparison**

A : Chroma filter cube “Qdot set with long pass emission filter“ (set 32013 blue: Exciter E460SPUVv2; red : Dichroic 475dcxru ; green : emitter E500lp)

B : Semrock filter (Bright line long-pass set. Blue : exciter FF02-435/40-21.8-D; red : FF510-Di02-22x29; green : FF01-500/LP-23.3-D) . In both the emission spectra of Qdot 525nm is shown shaded green

C & D : comparison between the exciter filter in the Chroma set FF02-435/40-21.8-D (black) and either FF02-435/40-21.8-D, from the Semrock set (**C**) or the FF01-417/60 (Semrock) (**D**) which were tested as replacement for the emission filter together within the other Chroma filters. The bleu & purple shaded areas represent [illegible] mrock have an average t[illegible]

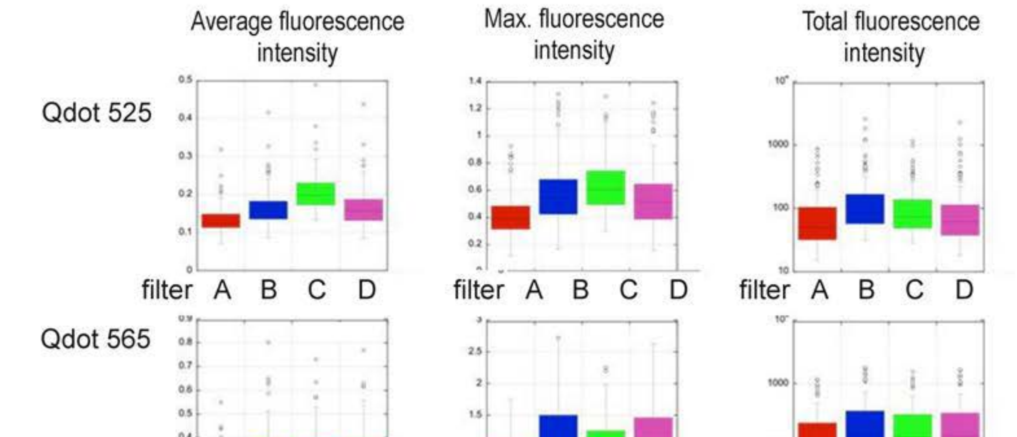

Figure B : Improvement of Filter cube - Box plot of the fluorescence intensity

Average, Max. & total fluorescence intensity (counts/s) in positive cells labelled with Qdots 525 565 585 605 625 655 &705, using 4 different filter combinations.

A : Chroma filter set 32013 : E460SPUVv2; 475dcxru ; E500lp

B : Semrock filter set : FF02-435/40-21.8-D; FF510-Di02-22x29 FF01-500/LP-23.3-D

C : FF02-435/40-21.8-D (Semrock), 475dcxru ; E500lp (both Chroma)

D : FF01-417/60 (Semrock), 475dcxru ; E500lp (both Chroma)

Qdot	525	565	585	605		625	655	705
	Comparing with Chroma Qdot filter - A							
B	better	better	Best	Best		Best	Best	Best
C	Best	better	better	better		better	better	better
D	better	better		better		better		

Table A : Improvement of Filter cube - Summary of the performance of each filter combination compared with set A
