# Supplementary material for: Choice of Illumination System & Fluorophore for Multiplex Immunofluorescence on FFPE Tissue Sections
Source: PLoS One. 2016 Sep 15;11(9):e0162419. doi: 10.1371/journal.pone.0162419 (PMC5025086; doi:10.1371/journal.pone.0162419)
Supplement: S1 Table — (PDF) [file pone.0162419.s007.pdf]

| Qdot | Light source | PE2 400nm | PE2 425nm | Metal Halide | Mercury | SOLA |
|---|---|---|---|---|---|---|
| 525 | Fluorescence Signal (mW) | 1.3344 | 1 | 0.66553 | 0.60772 | 0.34471 |
| | Excitation Light Noise (mW) | 0.24414 | 1 | 4396.4 | 4068 | 6418.7 |
| | Autofluorescence Noise (mW) | 0.99191 | 1 | 0.48742 | 0.51067 | 0.36511 |
| | Signal-to-Noise Ratio | 1.3452 | 1 | 1.3647 | 1.1895 | 0.94313 |
| 565 | Fluorescence Signal (mW) | 1.317 | 1 | 0.63069 | 0.59774 | 0.35309 |
| | Excitation Light Noise (mW) | 0.24414 | 1 | 4396.4 | 4068 | 6418.7 |
| | Autofluorescence Noise (mW) | 0.99191 | 1 | 0.48742 | 0.51067 | 0.36511 |
| | Signal-to-Noise Ratio | 1.3278 | 1 | 1.2933 | 1.1699 | 0.96611 |
| 585 | Fluorescence Signal (mW) | 1.2922 | 1 | 0.5994 | 0.57693 | 0.3469 |
| | Excitation Light Noise (mW) | 0.24414 | 1 | 4396.4 | 4068 | 6418.7 |
| | Autofluorescence Noise (mW) | 0.99191 | 1 | 0.48742 | 0.51067 | 0.36511 |
| | Signal-to-Noise Ratio | 1.3028 | 1 | 1.2291 | 1.1292 | 0.94919 |
| 605 | Fluorescence Signal (mW) | 1.2254 | 1 | 0.57997 | 0.56821 | 0.34615 |
| | Excitation Light Noise (mW) | 0.24414 | 1 | 4396.4 | 4068 | 6418.7 |
| | Autofluorescence Noise (mW) | 0.99191 | 1 | 0.48742 | 0.51067 | 0.36511 |
| | Signal-to-Noise Ratio | 1.2353 | 1 | 1.1893 | 1.1122 | 0.94714 |
| 625 | Fluorescence Signal (mW) | 1.1645 | 1 | 0.55437 | 0.38195 | 0.3433 |
| | Excitation Light Noise (mW) | 0.24414 | 1 | 4396.4 | 4068 | 6418.7 |
| | Autofluorescence Noise (mW) | 0.99191 | 1 | 0.48742 | 0.51067 | 0.36511 |
| | Signal-to-Noise Ratio | 1.174 | 1 | 1.1367 | 0.74762 | 0.93932 |
| 655 | Fluorescence Signal (mW) | 1.1908 | 1 | 0.56833 | 0.41734 | 0.34619 |
| | Excitation Light Noise (mW) | 0.24414 | 1 | 4396.4 | 4068 | 6418.7 |
| | Autofluorescence Noise (mW) | 0.99191 | 1 | 0.48742 | 0.51067 | 0.36511 |
| | Signal-to-Noise Ratio | 1.2005 | 1 | 1.1654 | 0.81689 | 0.94722 |
| 705 | Fluorescence Signal (mW) | 1.2069 | 1 | 0.576 | 0.56144 | 0.34652 |
| | Excitation Light Noise (mW) | 0.24414 | 1 | 4396.4 | 4068 | 6418.7 |
| | Autofluorescence Noise (mW) | 0.99191 | 1 | 0.48742 | 0.51067 | 0.36511 |
| | Signal-to-Noise Ratio | 1.2168 | 1 | 1.1811 | 1.0989 | 0.94818 |

**Table : Comparison of theoretical fluorescence parameters using semrocksearchlight.com.**
Filter Set : QDLP-C ; Exciter : FF01-405/150 ; Emitter : FF01-500/LP ; Dichroic : FF510-Di02 ; Light Source Power (mW) : 100 ; Numerical Aperture : 0.5 ; Index of Refraction : 1 ; Reflected Excitation Light Factor : 0.07 ; Transmitted Excitation Light Factor : 0.95 ; Fluorophore Optical Depth (M cm) : 1.00E-12 ; Fluorophore DMAC (M-1cm-1) : 80000 ; Fluorophore Quantum Yield : 0.5 ; Autofluorescence Light Factor : 0.1
Selected Light sources are : LED 400nm : 400 LED Single Wavelength CoolLED ; LED 425 : 425 LED Single Wavelength CoolLED; Metal Halide : Lamp Metal Halide - 120mW Lumen Dynamics X-Cite ; Mercury : Lamp Mercury Arc - Measured Semrock ; SOLA : LED Broadband Lumencor SOLA light engine.
