# Supplementary material for: Choice of Illumination System & Fluorophore for Multiplex Immunofluorescence on FFPE Tissue Sections
Source: PLoS One. 2016 Sep 15;11(9):e0162419. doi: 10.1371/journal.pone.0162419 (PMC5025086; doi:10.1371/journal.pone.0162419)
Supplement: S2 Table — (PDF) [file pone.0162419.s008.pdf]

Choosing the objective with the best NA (numerical aperture)(1)
The NA of an objective measure its resolving power and its ability to gather light, and the best possible objective should be chosen for quantification studies.
We compared 2 x40 objectives (NA 0.95 70.11 0-23/FN26.5 versus NA0.75) for the quality of the image obtained, the characteristics in term of efficiency to gather light between 500 & 720 nm (spectra on captured images) and the ability to gather light. Exposure time for capture of the images was determined at the peak of emission of the individual Qdots using the nuance autoexposure which prevent saturation of pixels by restricting the exposure time to a 70 % of the dynamic range of pixel values. The cubes of images were then captured between 500 & 720nm. There was no difference in term of the spectra of capture images between the 2 objectives (not shown). The quality of the images was very similar, but the time for exposure were 1.5 to 2.9 times faster with the objective with 0.95 aperture.
Possible photobleaching was checked by re-autoexposing the image after capture (The autoexposure is very fast, but illumination during the capture can take up to 1 minute : time to capture 45 images plus time to manually switch on and off the light - Some photobleaching occurred for Q dots 605 655 & 705 (S Prost Manuscript PONE-D-16-16178 ; under review)

	Autoexposure times at peak of emission in chronological order					
NA	0.75	0.95	0.75	0.75	0.95	0.95
Qdot	Initial autoexposure	Initial autoexposure	autoexposure + capture	autoexposure check after capture	autoexposure + capture	autoexposure check after capture
525	140.43	59.99	138.98		59.46	
565	123.12	49.43	119.04	118.74	50.08	50.50
585	218.32	86.20	220.56	212.10	80.22	80.11
**605**	**497.87**	**328.86**	**549.13**	**751.50**	**278.42**	**282.13**
625	51.28	22.95	50.660	50.93	22.30	22.05
**655**	**94.51**	**65.17**	**177.87**	**182.26**	**71.42**	**73.48**
**705**	**301.28**	**160**	**330.67**	**452.67 (*536.94)**	**292.45**	**300.21**

*check with 0.75 NA objective after "autoexposure check after capture" with the 0.95 NA objective
All exposure are given in ms
A cube of images taken every 5nm between 500 and 720nm was capture for each Qdot stain ie 45 images per cubes.

Table: Improvement of Objectives

1. Rottenfusser RW, E.E., Davidson. M.W. Numerical aperture and resolution http://zeiss-campus.magnet.fsu.edu [Available from: http://zeiss-campus.magnet.fsu.edu/articles/basics/resolution.html.
